# Supplementary material for: Is self-screening for 'at risk of malnutrition' feasible in a home setting?
Source: PLoS One. 2024 Apr 16;19(4):e0299305. doi: 10.1371/journal.pone.0299305 (PMC11020964; doi:10.1371/journal.pone.0299305)
Supplement: S1 Table — Abbreviations: BMI: body mass index, FS: final screening, HP: healthcare professionals, IS: initial screening, NRS: Nutritional risk screening P: patients. (PDF) [file pone.0299305.s001.pdf]

Department*	Preop_screening_HF	Age**	Male	CompletedScreening_HI	IS_LowBMI_H
1,00	0,00	5	0	1	0
1,00	0,00	4	0	1	0
1,00	0,00	5	1	1	0
1,00	0,00	4	0	1	0
1,00	0,00	5	1	1	0
1,00	1,00	3	0	1	0
1,00	0,00	2	0	1	0
1,00	0,00	5	1	1	0
1,00	0,00	7	0	1	1
1,00		5	0	0	
1,00	0,00	5	0	1	0
1,00	1,00	5	0	1	1
1,00	0,00	5	0	1	0
1,00	1,00	4	0	1	0
1,00	1,00	2	0	1	0
1,00	1,00	5	1	1	0
1,00	0,00	5	0	1	0
1,00	0,00	3	1	1	0
1,00	0,00	2	0	1	0
1,00		3	1	0	
1,00		4	0	0	
1,00	0,00	5	1	1	0
1,00	0,00	6	1	1	0
1,00	0,00	6	1	1	0
1,00	1,00	1	1	0	1
1,00	1,00	5	1	1	0
1,00	1,00	4	0	1	0
1,00	1,00	5	1	1	0
1,00		5	0	0	
1,00	1,00	5	1	1	0
1,00		5	1	0	
1,00	0,00	6	1	1	0
1,00		3	0	0	
1,00		5	1	0	
1,00		3	1	0	
1,00		5	1	0	
1,00		6	0	1	1
2,00		5	1	0	
2,00		7	1	0	
2,00		6	0	0	
2,00		4	1	0	
2,00	0,00	6	1	0	
2,00		1	0	0	
2,00	0,00	6	0	1	0
2,00		4	0	0	
2,00		5	1	0	
2,00		5	1	0	
2,00		3	0	0	
2,00	0,00	6	1	1	0
2,00	1,00	7	0	1	0
2,00		5	0	0	
2,00		4	1	1	0
2,00	1,00	4	1	1	0
2,00	1,00	6	1	1	0
2,00		4	1	1	0

2,00		5	1	0	
2,00		6	1	0	
2,00		6	1	0	
2,00	1,00	5	0	1	0
3,00		6	0	0	
3,00		4	0	0	
3,00		5	0	0	
3,00		4	0	0	
3,00		6	0	0	
3,00		5	0	0	
3,00		4	0	0	
3,00		5	0	0	
3,00		3	0	0	
3,00		4	0	0	
3,00		3	0	0	
3,00		3	0	0	
3,00		5	0	0	
3,00		4	0	0	
3,00		5	0	0	
3,00		5	0	0	
3,00		2	0	0	
3,00		5	0	0	
3,00		5	0	0	
3,00		4	0	0	
3,00		5	0	0	
3,00		5	0	0	
3,00		4	0	0	
3,00		5	0	0	
3,00		3	0	0	
3,00		6	0	0	
3,00		4	0	0	
3,00		6	0	0	
3,00		5	0	0	
3,00		4	0	0	
3,00		5	0	0	
3,00		5	0	0	
3,00		5	1	0	
3,00		4	0	0	
3,00		5	0	0	
3,00		4	0	0	
3,00		6	0	0	
3,00	0,00	4	0	1	0
3,00		5	0	0	
3,00		5	0	0	
3,00		4	0	0	
3,00		6	0	0	
3,00		5	0	0	
3,00		2	1	0	
3,00		6	0	0	
4,00	1,00	3	0	1	0
4,00	1,00	3	1	1	0
4,00		3	0	0	
4,00		3	0	0	
4,00		1	0	0	
4,00	0,00	1	1	1	0
4,00		6	1	0	

4,00		7	1	1	0
4,00	1,00	4	0	1	0
4,00		4	1	0	
4,00		6	0	0	
4,00	1,00	5	1	1	0
4,00	0,00	4	0	1	0
4,00		1	1	0	
4,00	1,00	3	1	0	0
4,00		1	0	0	
4,00	1,00	1	0	1	0
4,00	1,00	1	1	1	0
4,00	0,00	3	0	1	0
4,00		3	0	0	
4,00	1,00	2	0	1	0
4,00		4	1	0	
4,00	1,00	4	1	1	0
4,00		4	1	0	
4,00	0,00	2	0	1	0
4,00	1,00	3	1	1	0
4,00	1,00	4	0	1	0
4,00	1,00	4	1	1	0
4,00		2	0	0	
4,00		3	1	0	
4,00		6	1	0	
4,00	1,00	6	1	1	0
4,00	1,00	3	0	1	0
4,00		5	0	0	
4,00	0,00	5	0	1	0
4,00		5	0	0	
4,00	1,00	6	1	1	0
4,00		5	0	0	
4,00	1,00	2	1	0	0
4,00	1,00	5	1	1	0
4,00		4	1	0	
4,00	1,00	4	0	1	0
4,00		4	0	0	
5,00	1,00	3	0	1	0
5,00	1,00	2	0	0	0
5,00	1,00	3	0	1	0
5,00	1,00	3	1	1	0
5,00	1,00	4	1	1	0
5,00	1,00	4	1	1	0
5,00	1,00	2	0	1	0
5,00	1,00	4	0	1	0
5,00	1,00	5	0	1	0
5,00	1,00	4	0	1	0
5,00	1,00	3	1	1	0
5,00	1,00	4	0	1	0
5,00	1,00	5	0	1	0
5,00	1,00	4	1	1	0
5,00	1,00	5	1	1	0
5,00	1,00	5	1	1	0
5,00	1,00	4	0	1	0
5,00	1,00	4	1	1	0
5,00	1,00	5	0	1	0
5,00	1,00	4	1	1	0

5,00	1,00	4	0	1	0
5,00	1,00	5	0	1	0
5,00	1,00	3	0	1	0
5,00	1,00	3	1	1	0
5,00	1,00	5	1	1	0
5,00	1,00	4	0	1	0
5,00	1,00	4	1	1	0
5,00	1,00	5	1	1	0
5,00	1,00	3	0	1	0
5,00	1,00	5	1	1	0
5,00		6	1	1	0
5,00		5	0	1	0
6,00	1,00	5	1	1	0
6,00	1,00	3	1	1	0
6,00	1,00	6	1	1	0
6,00	1,00	6	1	1	0
6,00	1,00	6	1	1	0
6,00		6	1	1	0
6,00	1,00	5	1	1	0
6,00	1,00	5	1	1	0
6,00	1,00	3	1	1	0
6,00	1,00	4	1	1	0
6,00	1,00	5	1	1	0
6,00	1,00	5	1	1	0
6,00	1,00	5	1	1	0
6,00	1,00	5	0	0	0
6,00	1,00	3	1	1	0
6,00	1,00	5	1	1	0
6,00	1,00	5	1	1	0
6,00	0,00	5	1	1	0
6,00	1,00	5	1	0	
6,00	1,00	3	1	1	0
6,00	1,00	5	1	1	0
6,00	1,00	5	1	1	0
6,00	1,00	4	1	1	0
6,00	1,00	5	1	1	0
6,00	1,00	6	0	1	0
6,00	1,00	5	0	1	0
6,00	1,00	6	1	1	0
6,00	1,00	2	1	1	0
6,00	1,00	5	1	1	0
6,00	1,00	3	1	1	0
6,00	1,00	5	0	1	0
6,00	1,00	5	1	1	0
6,00	1,00	5	1	1	0
6,00	1,00	6	0	1	0
6,00	1,00	5	1	1	0
6,00	1,00	5	0	1	0

Abbrevations: **BMI:** body mass index, **FS:** final screening, **HP:** healthcare professionals, **IS:** initial screeni

*Departments: 1: Gastrointestinal, 2: Førde central hospital, 3: Breast and endocrine, 4: Ear, nose, throat/
**Age: 1: 18-29y, 2: 30-39y, 3: 40-49y, 4: 50-59y, 5: 60- 69y, 6: 70-79y, 7: 80-89y

IS_ReducedBodyWeight_	IS_ReducedDietaryIntake_HP	IS_CriticalIll_HP	IS_Yes_HP
0	0	0	0,00
0	0	0	0,00
0	1	0	1,00
0	0	0	0,00
1	1	0	1,00
0	0	0	0,00
1	1	0	1,00
0	0	0	0,00
0	1	0	1,00
0	0	0	0,00
0	0	0	1,00
0	1	0	1,00
0	0	0	0,00
1	0	0	1,00
0	0	0	0,00
0	0	0	0,00
0	1	0	1,00
0	0	0	0,00
0	0	0	0,00
0	0	0	0,00
1	1	0	1,00
1	0	0	1,00
1	0	0	1,00
0	0	0	0,00
0	0	0	0,00
1	1	1	1,00
0	1	0	1,00
1	0	0	1,00
1	0	0	1,00
0	0	0	0,00
0	0	1	0,00
0	0	0	0,00
1	0	0	1,00
0	0	0	0,00
0	0	0	0,00

0	0	0	0,00
0	1	0	1,00
0	0	0	0,00
0	0	0	0,00
0	0	0	0,00

0	0	0	0,00
0	0	0	0,00
0	0	0	0,00
0	0	0	0,00
	0	0	
0	0	0	0,00
0	0	0	0,00
0	0	0	0,00
0	0	0	0,00
0	0	0	0,00
0	0	0	0,00
0	0		0,00
0	0	0	0,00
0	0	0	0,00
0	0	0	0,00
1	0	0	1,00
0	0	0	0,00
0	0	0	0,00
1	0	0	1,00
0	0	0	0,00
0	0	0	0,00
0	0	0	0,00
0	1	0	1,00
1	0	0	1,00
0	0	0	0,00
0	0	0	0,00
0	0	0	0,00
0	0	0	0,00
0	0	0	0,00
0	0	0	0,00
0	0	0	0,00
0	0	0	0,00
0	0	0	0,00
0	0	0	0,00
0	0	0	0,00
0	0	0	0,00
0	0	0	0,00
0	0	0	0,00
0	0	0	0,00
0	0	0	0,00
0	0	0	0,00

0	0	0	0,00
0	0	0	0,00
0	0	0	0,00
0	0	0	0,00
0	0	0	0,00
0	0	0	0,00
0	0	0	0,00
0	0	0	0,00
0	0	0	0,00
0	0	0	0,00
0	0	0	0,00
0	0	0	0,00
0	0	0	0,00
0	0	0	0,00
0	0	0	0,00
0	0	0	0,00
0	0	0	0,00
0	0	0	0,00
0	0	0	0,00
0	0	0	0,00
0	0	0	0,00
0	0	0	0,00
0	0	0	0,00
0	0	0	0,00
0	0	0	0,00
1	0	0	1,00
0	0	0	0,00
0	0	0	0,00
0	0	0	0,00
0	0	0	0,00
0	0	0	0,00
0	0	0	0,00
0	0	0	0,00
0	0	0	0,00
0	0	0	0,00
0	0	0	0,00
0	0	0	0,00
0	0	0	0,00
0	0	0	0,00
0	0	0	0,00
0	0	0	0,00
0	0	0	0,00
0	0	0	0,00
0	0	0	0,00
0	0	0	0,00
0	0	0	0,00
0	0	0	0,00

ing, **P:** patients,

/Maxillo-Facial, 5: Neuro, 6:Cardio-Thoracic

FS_NutritionalRequirements_HP	FS_SeverityDisease_HP	AgeScore	AtRiskMalnutrition_HP
0	1	0	0
0	1	0	0
1	1	0	0
0	1	0	0
1	1	0	0
			0
3	1	0	1
1	0	0	0
0	1	1	0
0	1	0	0
0	0	0	0
2	1	0	1
			0
0	0	0	0
0	1		0
0	1	0	0
1	1		0
	1		0
0	0		0
			0
2	1	1	1
0	0		0
			0
			0
0	1	0	0
1	1	1	1
0	1	1	0
0	0	1	0
0	1	1	0
0	1	1	0
		0	0
0	0	0	0
		1	0
		0	0

1	1	0	0
1	1	0	0
			0
			0
			0

0	1	1	0
	1		0
			0
			0
			0
			0
			0
			0
			0
			0
			0
			0
			0
			0
0	0	0	0
			0
		1	0
			0
			0
			0
0	1	0	0
			0
			0
			0
			0
			0
			0
			0
			0
			0
			0
			0
			0
			0
			0
			0
			0
			0

1		0
		0
		0
		0
		0
		0
		0
		0
		0
		0
		0
		0
		0
		0
		0
		0
		0
		0
		0
		0
		0
		0
		0
		0
		0
	0	
		0
		0
		0
		0
		0
		0
		0
		0
	0	0
	1	0
		0
	1	0
	0	0
	0	0
		0
	0	0
	0	0
		0
		0
		0
		0

BMI_P	IS_LowBMI_	IS_ReducedBodyWeight_	IS_ReducedDietaryIntake_P
21,97	0,00	0,00	0,00
27,22	0,00		0,00
28,78	0,00	0,00	0,00
26,81	0,00		0,00
26,06	0,00	1,00	0,00
35,14	0,00	0,00	1,00
27,34	0,00	1,00	1,00
24,86	0,00	0,00	0,00
19,29	1,00	0,00	0,00
25,15	0,00	1,00	1,00
22,24	0,00	0,00	0,00
18,75	1,00	1,00	1,00
24,68	0,00	0,00	0,00
30,07	0,00	0,00	0,00
21,71	0,00	0,00	0,00
23,18	0,00	0,00	0,00
30,09	0,00		1,00
35,98	0,00	0,00	0,00
22,65	0,00	0,00	0,00
23,45	0,00	1,00	0,00
31,38	0,00		0,00
			0,00
25,80	0,00	0,00	0,00
30,69	0,00	1,00	0,00
18,03	1,00	1,00	0,00
26,78	0,00	1,00	0,00
30,10	0,00	0,00	1,00
24,57	0,00	0,00	0,00
29,41	0,00	0,00	0,00
33,08	0,00	1,00	1,00
25,25	0,00		0,00
25,76	0,00	0,00	0,00
35,14	0,00	0,00	0,00
30,54	0,00	1,00	0,00
25,54	0,00	0,00	0,00
23,99	0,00		0,00
20,20	1,00		1,00
29,05	0,00	1,00	0,00
25,83	0,00		1,00
27,89	0,00		0,00
20,43	1,00		1,00
24,68	0,00	0,00	0,00
27,89	0,00		0,00
24,31	0,00	1,00	0,00
20,03	1,00		1,00
			0,00
28,70	0,00	0,00	0,00
41,73	0,00	1,00	1,00
23,81	0,00	0,00	0,00
38,26	0,00	0,00	0,00
34,58	0,00	1,00	0,00
33,24	0,00	1,00	0,00
30,79	0,00	1,00	1,00
29,32	0,00		0,00

26,87	0,00	0,00	0,00
54,63	0,00	1,00	0,00
26,53	0,00	0,00	0,00
26,18	0,00	0,00	0,00
25,51	0,00		0,00
22,31	0,00	0,00	0,00
27,28	0,00	0,00	0,00
37,55	0,00	1,00	0,00
25,83	0,00		0,00
25,22	0,00	0,00	0,00
28,01	0,00		0,00
20,75	0,00	1,00	0,00
22,68	0,00	1,00	0,00
25,56	0,00	0,00	0,00
23,12	0,00	0,00	0,00
			0,00
24,62	0,00	1,00	0,00
19,84	1,00	0,00	1,00
29,00	0,00	0,00	0,00
22,01	0,00	1,00	1,00
24,61	0,00		0,00
24,84	0,00	1,00	0,00
27,55	0,00	1,00	0,00
31,98	0,00	0,00	1,00
32,53	0,00	0,00	0,00
21,80	0,00	0,00	0,00
20,48	1,00	1,00	1,00
35,16	0,00	0,00	1,00
28,39	0,00	1,00	0,00
32,21	0,00		0,00
30,80	0,00		0,00
19,44	1,00	1,00	0,00
36,63	0,00	0,00	0,00
23,84	0,00		1,00
29,76	0,00	1,00	0,00
30,07	0,00	1,00	0,00
28,37	0,00	0,00	0,00
22,96	0,00		0,00
		0,00	0,00
27,85	0,00	0,00	1,00
20,54	0,00		0,00
29,30	0,00		0,00
28,47	0,00	1,00	0,00
27,64	0,00		0,00
28,34	0,00	0,00	1,00
26,86	0,00		0,00
30,12	0,00		0,00
27,66	0,00	1,00	1,00
29,68	0,00	0,00	0,00
25,59	0,00		1,00
26,61	0,00	0,00	0,00
26,37	0,00	1,00	0,00
23,45	0,00	0,00	0,00
			0,00

23,46	0,00	1,00	0,00
27,76	0,00	0,00	0,00
22,99	0,00	0,00	0,00
26,12	0,00	0,00	0,00
29,70	0,00	0,00	0,00
35,92	0,00	0,00	0,00
18,82	1,00		
33,18	0,00	0,00	0,00
20,69	0,00		0,00
23,33	0,00	0,00	0,00
28,58	0,00	0,00	0,00
35,76	0,00	0,00	0,00
33,23	0,00	0,00	0,00
35,18	0,00	0,00	0,00
34,44	0,00	0,00	0,00
26,45	0,00	0,00	0,00
26,47	0,00	0,00	0,00
28,91	0,00	0,00	0,00
		1,00	0,00
37,65	0,00	1,00	1,00
32,33	0,00	0,00	0,00
24,38	0,00	1,00	0,00
32,00	0,00	0,00	0,00
32,11	0,00	1,00	1,00
28,72	0,00		0,00
29,71	0,00	0,00	0,00
39,54	0,00	0,00	0,00
24,88	0,00	0,00	0,00
27,99	0,00		0,00
24,06	0,00	1,00	0,00
30,39	0,00	0,00	0,00
27,77	0,00	1,00	1,00
24,38	0,00		0,00
20,31	1,00	1,00	0,00
28,13	0,00	0,00	0,00
24,17	0,00	1,00	1,00
35,76	0,00	1,00	1,00
24,97	0,00	0,00	0,00
			0,00
27,77	0,00	0,00	0,00
29,07	0,00	0,00	0,00
25,14	0,00	0,00	1,00
22,06	0,00		
23,71	0,00	0,00	0,00
27,70	0,00		0,00
		0,00	0,00
34,47	0,00		0,00
24,84	0,00	0,00	0,00
27,43	0,00	0,00	0,00
25,95	0,00	0,00	0,00
27,14	0,00	0,00	0,00
30,85	0,00		0,00
			0,00

41,23	0,00	0,00	0,00
22,59	0,00	0,00	0,00
25,51	0,00	0,00	0,00
28,09	0,00	0,00	0,00
23,46	0,00		0,00
36,52	0,00		0,00
24,00	0,00	1,00	0,00
26,85	0,00	0,00	0,00
29,73	0,00	0,00	0,00
25,59	0,00	0,00	0,00
			0,00
33,66	0,00		0,00
23,67	0,00	0,00	1,00
29,73	0,00		0,00
23,94	0,00	1,00	0,00
28,41	0,00	0,00	0,00
23,15	0,00	0,00	1,00
22,41	0,00	1,00	0,00
24,84	0,00	0,00	0,00
31,80	0,00	1,00	0,00
24,72	0,00	0,00	0,00
30,27	0,00		1,00
28,70	0,00		0,00
38,76	0,00		0,00
24,16	0,00		0,00
23,45	0,00	0,00	1,00
29,71	0,00		0,00
29,40	0,00	0,00	1,00
26,20	0,00	1,00	1,00
25,18	0,00	0,00	0,00
25,51	0,00	0,00	0,00
28,91	0,00		0,00
37,98	0,00	0,00	0,00
29,37	0,00	0,00	0,00
30,30			
27,24	0,00	0,00	0,00
27,22	0,00	0,00	0,00
23,67	0,00		0,00
22,69	0,00	0,00	0,00
37,75	0,00		0,00
27,17	0,00	1,00	0,00
22,89	0,00	0,00	0,00
29,37	0,00		0,00
33,67	0,00	0,00	0,00
22,49	0,00		1,00
25,43	0,00	0,00	0,00
23,31	0,00		0,00

FS_NutritionalRequirements_P	Sykdomsscore1_NI	BeliveToBeAtRiskOfMalnutrition_P
1	0,00	0
1	0,00	0
1	0,00	0
1	1,00	0
1	1,00	0
2	1,00	0
2	0,00	0
1	0,00	0
1	0,00	0
3	0,00	0
1	0,00	0
2	0,00	1
1	0,00	0
1	0,00	0
1	0,00	0
1	0,00	0
3	1,00	0
1	0,00	0
1		0
1	0,00	0
1	0,00	0
1	0,00	0
1	0,00	0
1	0,00	0
1	1,00	1
1	1,00	0
3	0,00	0
1	1,00	0
1	0,00	0
3	1,00	0
1	0,00	0
1	0,00	0
1	0,00	0
1	0,00	0
1	0,00	0
1	1,00	0
2	0,00	1
1	0,00	0
4	0,00	0
1	0,00	0
4	1,00	1
1	0,00	0
1	0,00	0
1	0,00	0
2	1,00	1
1	0,00	0
1	0,00	0
3	0,00	0
1	0,00	0
1	1,00	0
1	0,00	0
1	0,00	0
2	0,00	0
1	0,00	0

1	0,00	0
1	0,00	0
1	0,00	0
1	1,00	0
1		0
1	0,00	0
1	0,00	0
1	0,00	0
1	0,00	0
1	0,00	0
1	0,00	0
1	0,00	0
1	0,00	0
1	0,00	0
1	1,00	0
1	0,00	0
1		0
2	1,00	0
1	0,00	0
2	1,00	1
1	0,00	0
1	0,00	0
1	1,00	1
3	0,00	0
1	0,00	0
1	0,00	0
2	0,00	0
2	0,00	0
1	0,00	0
1	0,00	0
1	0,00	1
1		
1	0,00	0
2	0,00	0
1	0,00	0
1	1,00	0
1	0,00	0
1	0,00	0
1	0,00	0
2	0,00	0
1	0,00	
1	0,00	0
1	0,00	0
1	0,00	0
3	0,00	0
1	0,00	0
1	1,00	0
2	1,00	0
1	0,00	0
2	0,00	0
1	0,00	0
1	0,00	0
1	0,00	0
1	0,00	0

1	1,00	1
1	0,00	0
1	1,00	0
1	1,00	0
1	0,00	0
1	0,00	0
	0,00	0
1	0,00	0
	0,00	0
1	0,00	0
1	0,00	0
1	1,00	0
1	1,00	0
1	1,00	0
1	0,00	0
1	0,00	0
1	1,00	0
1	0,00	0
1	0,00	0
1	0,00	0
2	1,00	0
1	0,00	0
1	0,00	0
1	1,00	0
2	0,00	0
1	0,00	0
1	1,00	0
1	1,00	0
1	0,00	0
1	0,00	0
1	0,00	0
1	0,00	0
3	0,00	0
1	0,00	0
1	0,00	0
1	1,00	0
2	1,00	0
2	0,00	0
1	0,00	0
1	0,00	0
1	0,00	0
1	0,00	0
2	0,00	0
1	0,00	0
1	0,00	0
1	0,00	0
1	1,00	0
1	0,00	0
1	0,00	0
1	1,00	0
1	0,00	0
1	0,00	0
1	0,00	0

1	0,00	0
1	0,00	0
1	0,00	0
1	0,00	0
1	0,00	0
1	0,00	0
1	0,00	0
1	0,00	0
1	0,00	0
1	0,00	0
1	0,00	0
1	0,00	0
3	0,00	0
1	0,00	0
1	0,00	0
1	0,00	0
4	0,00	0
1	0,00	0
1	0,00	0
1	0,00	0
1	0,00	0
2	0,00	0
1	0,00	0
1	1,00	0
1	0,00	0
2	1,00	0
1	0,00	0
3	0,00	0
2	0,00	0
1	0,00	0
1	0,00	0
1	0,00	0
1	0,00	0
1	0,00	0
		0
1	0,00	0
1	0,00	
1	0,00	0
1	0,00	0
1	0,00	0
1	0,00	0
1	0,00	0
1	0,00	0
1	0,00	0
2	0,00	0
1	0,00	0
1	0,00	0

AtRiskOfMalnutrition_P
0,00
0,00
0,00
0,00
0,00
0,00
0,00
0,00
0,00
0,00
0,00
0,00
0,00
0,00
0,00
1,00
0,00
0,00
0,00
0,00
1,00
0,00
0,00
0,00
0,00
1,00
0,00
0,00
0,00
0,00
0,00
0,00
0,00
1,00
1,00
0,00
1,00
0,00
0,00
0,00
0,00
0,00
0,00
0,00
0,00

0,00
0,00
0,00
0,00

0,00
0,00
0,00

0,00

0,00
0,00
0,00
0,00

1,00
0,00
0,00

0,00
0,00
0,00
0,00
0,00
0,00
0,00
0,00
0,00

0,00
0,00
0,00
0,00
0,00
0,00

0,00
0,00

0,00

0,00
0,00

1,00
0,00
0,00
0,00
0,00
0,00

0,00
0,00
0,00
0,00
0,00
0,00
0,00
0,00

0,00
0,00
0,00
0,00
0,00
0,00
0,00

0,00
0,00
0,00
1,00
0,00
0,00
0,00
1,00

0,00
0,00
0,00

0,00
0,00
0,00

0,00
0,00
0,00
0,00
0,00

0,00
0,00
0,00

0,00

0,00
0,00
0,00
0,00
0,00
0,00

0,00
0,00
0,00
0,00

0,00
0,00
0,00
0,00
0,00

0,00

0,00
1,00
0,00
0,00
0,00
0,00
0,00
0,00

0,00
0,00

0,00
0,00
0,00
0,00

0,00
0,00
0,00
0,00
0,00

0,00

0,00
0,00

0,00
0,00
0,00
