# Supplementary material for: Is self-screening for 'at risk of malnutrition' feasible in a home setting?
Source: PLoS One. 2024 Apr 16;19(4):e0299305. doi: 10.1371/journal.pone.0299305 (PMC11020964; doi:10.1371/journal.pone.0299305)
Supplement: S2 File — The current study was a part of a development and validation Patient Safety Checklist (PASC) (ClinicalTrails.gov: NCT03105713). (PDF) [file pone.0299305.s003.pdf]

A copy from ClinicalTrails.gov

Trial record **1 of 1** for: NCT03105713

Development and Implementation of Patient Safety Checklists Before, During and After In-hospital Surgery (PASC)

ClinicalTrials.gov Identifier: NCT03105713

Recruitment Status : Recruiting
First Posted : April 10, 2017
Last Update Posted : March 18, 2022
See **Contacts and Locations**

View this study on Beta.ClinicalTrials.gov

Sponsor:
Haukeland University Hospital **Collaborators:**
Helse Vest
Western Norway University of Applied Sciences
University of Bergen
The Research Council of Norway
Information provided by (Responsible Party):
Haukeland University Hospital

Study Details

No Results Posted

Study Description

Brief Summary:
Building on the Norwegian Patient Safety Program's target areas, the Patients' Surgical Checklist (PASC) will empower surgical patients to become more involved in their own safety and contribute to preventive safety measures. A safety checklist for patients to use has been developed and validated for use in surgical patients. In a Stepped Wedge Cluster RCT effects of patients using their own checklists to avoid preventable patient harm are examined. The project will re-use existing health and personal data collected from patient records and patient reported data as outcome measures. A consortium of all relevant stakeholders and users participate: two hospitals with seven surgical clusters, patient representatives, representatives of general practitioners, and interdisciplinary in-hospital professionals. The important project partners are information and communications technology companies (Helse-Vest IKT and CheckWare service delivery), general practitioners, and national and international research partners leading in the field of patient safety, implementation science and health economics.

Condition or disease	Intervention/treatment	Phase
Surgery<br><br>Safety Issues<br><br>Complication	Other: Patient Safety Checklist	Not Applicable

Patient Safety<br><br>Health Literacy<br><br>Health Economics		

Detailed Description:

The PASC consist of measures that enable patients to optimize their own health prior to surgery and for discharge from hospital. The checklist addresses risk areas as preoperative information and preparations, post-operative information, and post-operative plans and follow-up. Pre-operative risk areas are contact information, medication safety, health status, optimizing health and nutritional status, dental status, comprehend critical information, preparation two weeks before surgery, communication with surgical ward, and discharge planning. Post-operative risk areas are prevention of complications, medication safety, activity restriction, and pain relief.

The checklist has been developed in cooperation with patients, patients' representatives, surgeons, general practitioners, ward doctors, nurses, pharmacists, clinical nutritionists, safety officers, hospital managers, information technology experts and the researchers. The intervention include paper and electronically versions of the checklist. Of eligible surgical wards, seven were randomly selected based on power calculation. All the invited wards agreed to participate. Surgical patients from these wards, in two Norwegian hospitals, will be invited to participate in the trial. Based on data from a validation and feasibility study of PASC, the power analysis suggest to include 38 patients per month (on average), per cluster over 20 months, as the lowest number of participants to detect a 5% (33.3% relative risk reduction). An intra-cluster-correlation at 0.05, and type I and type II error at 0.05 and 0.20, respectively, were assumed.

The outcomes of this study are primarily patient outcomes (morbidity and mortality). The study further assess outcomes on nutritional status (PG-SGA-SH form), implementation (acceptability, appropriateness and feasibility survey), health economic (EQ-5D-3L) data, and health literacy (HLQ), from surveys and forms.

Based on power calculation, 350 questionnaires will be distributed in each arm of the trial (baseline and internvention).

Focus group interviews with content analysis will be applied to assess patients and health care personnel's experiences with patients' use of PASC.

Study Design

Study Type :	Interventional (Clinical Trial)
Estimated Enrollment :	5320 participants
Allocation:	Randomized
Intervention Model:	Crossover Assignment
Intervention Model Description:	Stepped Wedge Cluster RCT is considered to align a cross-over design.
Masking:	Double (Care Provider, Outcomes Assessor)

Masking Description:	Care Providers are masked for patients receiving the intervention and for outcomes.<br><br>Outcome sssessors are masked for patients receiving the intervention.
Primary Purpose:	Health Services Research
Official Title:	Development and Implementation of Surgical Safety Checklists for Patients to Use<br>Before Admission, Before Discharge and After Discharge (PASC) - a Stepped Wedge Cluster Randomized Controlled Trial
Actual Study Start Date :	November 1, 2021
Estimated Primary Completion Date :	August 31, 2023
Estimated Study Completion Date :	August 31, 2025

Resource links provided by the National Library of Medicine

MedlinePlus related topics: Patient Safety Safety

U.S. FDA Resources

Arms and Interventions

Arm	Intervention/treatment
Experimental: Patient Safety Checklist Intervention<br><br>The intervention to be administered is a patient safety checklist (two parts) for patients to be performed on paper or electronically: a) before admission to hospital, and b) under hospital stay (discharge)	Other: Patient Safety Checklist<br><br>The intervention is developed, validated and to be implemented in seven surgical clusters in two hospitals, in a stepped wedge cluster randomized controlled trial of the PASC for patients to use.
No Intervention: Controls<br><br>Patients do not receive the safety checklist intervention. Care as usual.	

Outcome Measures

Primary Outcome Measures :

1. Number of complications associated with the participants surgery [Time Frame: Up to 30 days]

   Total numbers of complications

2. Number of complications within respiratory system [Time Frame: Up to 30 days]

   Number of respiratory complications

3. Number of complications within cardio-thoracic system [Time Frame: Up to 30 days]

   Number of cardio-thoracic complications

4. Number of infections [Time Frame: Up to 30 days]
   Numbers of infections

5. Number of nervous system complications [Time Frame: Up to 30 days]

   Number of nervous system complications

6. Volume of bleeding associated with operation [Time Frame: Up to 30 days]

   Volume of bleedings in mL

7. Number of embolism associated with the hospital stay [Time Frame: Up to 30 days]

   Number of embolies

8. Number of mechanical implant complications [Time Frame: Up to 30 days]

   Number of mechanical implant complications

9. Number of re-operations [Time Frame: Up to 30 days]

   Number of re-operations

10. Number of re-admissions [Time Frame: Up to 30 days]

    Number of re-admissions

Secondary Outcome Measures :

1. Number of deaths associated with surgery [Time Frame: Up to 90 days]

   Total numbers of deaths

2. Rate of patient scores on Health Literacy Questionaire [Time Frame: Up to 3 months post discharge]

   Mean scores of HLQ and EQ5D surveys

3. Rate of patient scores on EQ5D [Time Frame: Up to 3 months]

   Mean scores of EQ5D

4. Rates of Checklist Implementation Survey scores [Time Frame: Up to 3 months post discharge]

   Mean scores on checklist implementation survey

Eligibility Criteria

Ages Eligible for Study: 18 Years and older (Adult, Older Adult) Sexes Eligible for Study: All

Accepts Healthy Volunteers: No

Criteria

Inclusion Criteria:

Elective surgery.

- Age over 18 years.
- Able to use Norwegian language.
- Patients must have had surgery 2-4 weeks before inclusion for the focus group interviews.

Exclusion Criteria:

- Must be cognitive able to use the checklist.
- Age under 18 years. Non-surgical procedures.

Information:
The Research Council of Norway's Project Bank description of the PASC project

Study Data/Documents: Study Protocol
Identifier: 2016/1102
Study protocol in English and other relevant information on this study can be accessed by

Responsible Party:	Haukeland University Hospital
ClinicalTrials.gov Identifier:	NCT03105713 History of Changes
Other Study ID Numbers:	REK Vest (2016/1102)
First Posted:	April 10, 2017 Key Record Dates
Last Update Posted:	March 18, 2022
Last Verified:	December 2021

Individual Participant Data (IPD) Sharing Statement:

Plan to Share IPD: No

Plan Description: REKVest has decided that data which are anonymous, where it d other scientists or scientific journals.

Studies a U.S. FDA-regulated Drug Product: No

Studies a U.S. FDA-regulated Device No

Keywords provided by Haukeland University Hospital:

Checklist
Patient Checklist
Surgery
Safety
Patient Involvement
Undernutrition
Implementation
Health literacy
Health economics
