# Supplementary material for: Is self-screening for 'at risk of malnutrition' feasible in a home setting?
Source: PLoS One. 2024 Apr 16;19(4):e0299305. doi: 10.1371/journal.pone.0299305 (PMC11020964; doi:10.1371/journal.pone.0299305)
Supplement: Attachment — Submitted filename: PONE-D-22-30022 - MANUSCRIPT.pdf [file pone.0299305.s005.pdf]

PLOS ONE

Surgical patients’ self-screening for “at risk of malnutrition” in a home setting – a feasibility study (ClinicalTrails.gov: NCTT03105713)

--Manuscript Draft--

Manuscript Number:	PONE-D-22-30022
Article Type:	Clinical Trial
Full Title:	Surgical patients’ self-screening for “at risk of malnutrition” in a home setting – a feasibility study (ClinicalTrails.gov: NCTT03105713)
Short Title:	Preoperatively self-screening for "at risk of malnutrition"
Corresponding Author:	Eli Skeie<br>Haukeland University Hospital<br>Bergen, NORWAY
Keywords:	Malnutrition, elective surgery, nutritional risk screening, self-screening
Abstract:	Introduction<br><br>Despite malnutrition being a well-known risk for postoperative complications, the lack of nutritional risk screening remains a challenge. The aim of this study was to investigate whether self-screening for nutritional risk prior to surgery in a home setting will increase amount of patients screened for nutritional risk, and secondly, to compare their screening results with healthcare professionals’ in-hospital assessment.<br><br>Materials and methods<br><br>This was a prospective study involving patients from six randomly selected surgical wards at two Norwegian hospitals as a part of a development and validation patient’s safety checklist study (PASC) (ClinicalTrails.gov: NCTT03105713). As part of the checklist, patients were preoperatively in a home-setting asked questions needed to identify “at risk of malnutrition” by the Nutritional Risk Screening tool (NRS 2002). The same screening tool was used as a standard routine to identify “at risk of malnutrition” by healthcare professionals at hospital. The interrater reliability between these results was investigated using Kappa Measure of Agreement and reported with Landis and Koch’s grading system (poor, slight, fair, moderate, substantial and almost perfect).<br><br>Results<br><br>Of the 215 surgical patients, 165 (76.7%) patients carried out the nutritional risk screening in a home setting, of whom 69 (32.1%) were not screened by a healthcare professional at hospital. At the hospital, 123 (57.2%) were screened for being “at risk of malnutrition” by healthcare professionals, and of these, 27 (12.6%) patients did not complete the self-screening. The degree of agreement between the answers of patients and healthcare workers to the NRS 2002 questions regarding low body mass index, weight loss and reduced food intake was almost perfect, moderate and slight, respectively. However, the degree of agreement regarding being “at risk of malnutrition” or not was poor.<br><br>Conclusions<br><br>Preoperatively self-screening in a home setting may be a feasible method to increase the number of elective surgical patients screened for being “at risk of malnutrition”.
Order of Authors:	Eli Skeie
	Kristin Harris
	Arvid Steinar Haugen
	Randi Julie Tangvik
	Stig Harthug

Powered by Editorial Manager® and ProduXion Manager® from Aries Systems Corporation

Additional Information:	
**Question**	**Response**
**Financial Disclosure**<br><br>Enter a financial disclosure statement that describes the sources of funding for the work included in this submission. Review the submission guidelines for detailed requirements. View published research articles from *PLOS ONE* for specific examples.<br><br>This statement is required for submission and **will appear in the published article** if the submission is accepted. Please make sure it is accurate.<br><br>**Unfunded studies**<br>Enter: *The author(s) received no specific funding for this work.*<br><br>**Funded studies**<br>Enter a statement with the following details:<br>• Initials of the authors who received each award<br>• Grant numbers awarded to each author<br>• The full name of each funder<br>• URL of each funder website<br>• Did the sponsors or funders play any role in the study design, data collection and analysis, decision to publish, or preparation of the manuscript?<br>• **NO** - Include this sentence at the end of your statement: *The funders had no role in study design, data collection and analysis, decision to publish, or preparation of the manuscript.*<br>• **YES** - Specify the role(s) played.<br><br>* typeset	ES received PhD-grant from the Western Norway Regional Health Authority Trust (grant number 912214). ASH received research grant from the Western Norway Regional Health Authority Trust (grant number HV1172).<br><br>https://helse-vest.no/en<br><br>KH received PhD grant from the Western Norway University of Applied Sciences (no grant number).<br><br>https://www.hvl.no/en/<br><br>The funders did not play any role in the study design, data collection and analysis, decision to publish, or preparation of the manuscript.
**Competing Interests**<br><br>Use the instructions below to enter a competing interest statement for this submission. On behalf of all authors, disclose any competing interests that could be perceived to bias this work—acknowledging all financial support and any other relevant financial or non-financial competing interests.	I have read the journal's policy and the authors of this manuscript have the following competing interests: ASH has previously received travel re-imbursement for representing the International Federation of Nurse Anaesthetists in the European Society of Anaesthesiology and Intensive Care's Patient Quality and Safety Committee. All the other authors have declared that no competing interests exist.

This statement is required for submission and **will appear in the published article** if the submission is accepted. Please make sure it is accurate and that any funding sources listed in your Funding Information later in the submission form are also declared in your Financial Disclosure statement.<br><br>View published research articles from *PLOS ONE* for specific examples.<br><br>**NO authors have competing interests**<br><br>Enter: *The authors have declared that no competing interests exist.*<br><br>**Authors with competing interests**<br><br>Enter competing interest details beginning with this statement:<br><br>*I have read the journal's policy and the authors of this manuscript have the following competing interests: [insert competing interests here]*<br><br>* typeset	
**Ethics Statement**<br><br>Enter an ethics statement for this submission. This statement is required if the study involved:<br><br>• Human participants<br>• Human specimens or tissue<br>• Vertebrate animals or cephalopods<br>• Vertebrate embryos or tissues<br>• Field research<br><br>Write "N/A" if the submission does not require an ethics statement.<br><br>General guidance is provided below. Consult the submission guidelines for detailed instructions. **Make sure that all information entered here is included in the**	The Regional Ethics Committee West for medical and health research and the data privacy ombudsman approved the study prior to start (Ref: 2016/1102/REC/west). All participants signed an informed consent form after receiving information about the study at recruitment, including that their participation in the study was voluntary and that they could withdraw without consequence at any time.

**Methods section of the manuscript.**<br><br>**Format for specific study types**<br><br>**Human Subject Research (involving human participants and/or tissue)**<br>• Give the name of the institutional review board or ethics committee that approved the study<br>• Include the approval number and/or a statement indicating approval of this research<br>• Indicate the form of consent obtained (written/oral) or the reason that consent was not obtained (e.g. the data were analyzed anonymously)<br><br>**Animal Research (involving vertebrate animals, embryos or tissues)**<br>• Provide the name of the Institutional Animal Care and Use Committee (IACUC) or other relevant ethics board that reviewed the study protocol, and indicate whether they approved this research or granted a formal waiver of ethical approval<br>• Include an approval number if one was obtained<br>• If the study involved *non-human primates*, add *additional details* about animal welfare and steps taken to ameliorate suffering<br>• If anesthesia, euthanasia, or any kind of animal sacrifice is part of the study, include briefly which substances and/or methods were applied<br><br>**Field Research**<br><br>Include the following details if this study involves the collection of plant, animal, or other materials from a natural setting:<br>• Field permit number<br>• Name of the institution or relevant body that granted permission	
**Data Availability**<br><br>Authors are required to make all data underlying the findings described fully available, without restriction, and from the time of publication. PLOS allows rare exceptions to address legal and ethical concerns. See the PLOS Data Policy and FAQ for detailed information.	Yes - all data are fully available without restriction

A Data Availability Statement describing where the data can be found is required at submission. Your answers to this question constitute the Data Availability Statement and **will be published in the article**, if accepted.<br><br>**Important:** Stating 'data available on request from the author' is not sufficient. If your data are only available upon request, select 'No' for the first question and explain your exceptional situation in the text box.<br><br>Do the authors confirm that all data underlying the findings described in their manuscript are fully available without restriction?	
**Describe where the data may be found in full sentences. If you are copying our sample text, replace any instances of XXX with the appropriate details.**<br><br>• If the data are **held or will be held in a public repository**, include URLs, accession numbers or DOIs. If this information will only be available after acceptance, indicate this by ticking the box below. For example: *All XXX files are available from the XXX database (accession number(s) XXX, XXX.*).<br>• If the data are all contained **within the manuscript and/or Supporting Information files**, enter the following: *All relevant data are within the manuscript and its Supporting Information files.*<br>• If neither of these applies but you are able to provide **details of access elsewhere**, with or without limitations, please do so. For example:<br><br>*Data cannot be shared publicly because of [XXX]. Data are available from the XXX Institutional Data Access / Ethics Committee (contact via XXX) for researchers who meet the criteria for access to confidential data.*<br><br>*The data underlying the results*	All relevant data are within the manuscript and its Supporting Information files.

Powered by Editorial Manager® and ProduXion Manager® from Aries Systems Corporation

presented in the study are available from (include the name of the third party and contact information or URL).<br>• This text is appropriate if the data are owned by a third party and authors do not have permission to share the data.<br><br>* typeset	
Additional data availability information:	Tick here if the URLs/accession numbers/DOIs will be available only after acceptance of the manuscript for publication so that we can ensure their inclusion before publication.

Powered by Editorial Manager® and ProduXion Manager® from Aries Systems Corporation

Manuscript

Click here to access/download;Manuscript;Manuscript_PlosOne_Skeie_new

Surgical patients’ self-screening for “at risk of malnutrition” in a home setting – a feasibility study (ClinicalTrails.gov: NCT03105713)

Eli Skeie[1*], Kristin Harris[2, 3, 4], Arvid Steinar Haugen[3, 4], Randi Julie Tangvik[3, 5], Stig Harthug[1, 6]

[1] Department of Research and Development, Haukeland University Hospital, Bergen, Norway;

[2] Department of Health and Caring Sciences, Western Norway University of Applied Sciences, Bergen, Norway;

[3] Department of Anaesthesia and Intensive Care, Haukeland University Hospital, Bergen, Norway;

[4] Department of Nursing and Health Promotion Acute and Critical Illness, Faculty of Health Sciences, OsloMet – Oslo Metropolitan University, Oslo, Norway;

[5] Mohn Nutrition Research Laboratory, Centre for Nutrition, Department of Clinical Medicine, University of Bergen, Bergen, Norway;

[6] Department of Clinical Science, University of Bergen, Bergen, Norway

* Corresponding author

The study was mainly funded through PhD-grants for ES and KH from the Western Norway Regional Health Authority Trust (grant number 912214) and the Western Norway University of Applied Sciences, respectively. In addition, ASH received research grant from the Western Norway Regional Health Authority Trust (HV1172). KH, ASH and RJT are affiliated with the PASC project funded by the Norwegian Council of Research (320475). Others were funded by their respectively departments.

Abstract

Introduction: Despite malnutrition being a well-known risk for postoperative complications, the lack of nutritional risk screening remains a challenge. The aim of this study was to investigate whether self-screening for nutritional risk prior to surgery in a home setting will increase amount of patients screened for nutritional risk, and secondly, to compare their screening results with healthcare professionals' in-hospital assessment.

Materials and methods: This was a prospective study involving patients from six randomly selected surgical wards at two Norwegian hospitals as a part of a development and validation Patient Safety Checklist (PASC) (ClinicalTrails.gov: NCT03105713). As part of the checklist, patients were preoperatively in a home-setting asked questions needed to identify "at risk of malnutrition" by the Nutritional Risk Screening tool (NRS 2002). The same screening tool was used as a standard routine to identify "at risk of malnutrition" by healthcare professionals at hospital. The interrater reliability between these results was investigated using Kappa Measure of Agreement and reported with Landis and Koch's grading system (*poor, slight, fair*, *moderate*, *substantial* and *almost perfect*).

Results: Of the 215 surgical patients, 165 (76.7%) patients carried out the nutritional risk screening in a home setting, of whom 69 (32.1%) were not screened by a healthcare professional at hospital. At the hospital, 123 (57.2 %) were screened for being "at risk of malnutrition" by healthcare professionals, and of these, 27 (12.6%) patients did not complete the self-screening. The degree of agreement between the answers of patients and healthcare workers to the NRS 2002 questions regarding low body mass index, weight loss and reduced food intake was *almost perfect*, *moderate* and *slight*, respectively. However, the degree of agreement regarding being "at risk of malnutrition" or not was *poor*.

Conclusions: Preoperatively self-screening in a home setting may be a feasible method to increase the number of elective surgical patients screened for being “at risk of malnutrition”.

Introduction

Malnutrition is an established risk factor for postoperative complications, and should thus be prevented and treated prior to surgery (1, 2). To identify patients who are diagnosed as malnourished or “at risk of malnutrition”, both European (3) and Norwegian (4) guidelines for preventing and treating malnutrition recommend nutritional risk screening at admission. For this purpose, the screening tool Nutrition Risk Screening 2002 (NRS 2002) (5) has been recommended for use by healthcare professionals on in-hospital surgical patients (3, 6). The nutritional risk screening and associated treatment should occur early in the patient care process (6), since the risk of malnutrition may worsen over time and negatively affect patient outcomes (7). Regarding elective surgery, the time between when surgery is booked and the actual surgical procedure may be suitable for detecting and starting to treat malnutrition. Thus, the most recent recommendations for perioperative nutrition from the European Society for Clinical Nutrition and Metabolism expert group recommend that patients be screened for being “at risk of malnutrition” at least 10 days before surgery (6). However, this recommendation may be difficult to follow since most elective patients are admitted at hospital at a time closer to the surgery.

The World Health Organization and European patient organizations endorse patient’s involvement in safety (8, 9), and it has been suggested that patients can screen themselves for being at “at risk of malnutrition” (10). Moreover, surgical patients and healthcare professionals have identified nutritional status to be a safety risk factor that should be included in a patient-driven surgical patient safety checklist (11). Previous studies have demonstrated that patients can successfully screen themselves in an outpatient setting for being “at risk of malnutrition” using the Patient-Generated Subjective Global Assessment Short Form (12), the Malnutrition Universal Screening Tool (MUST) (10) and the Malnutrition Screening Tool (MST) (13). However, there is a lack of knowledge and a need

for studies investigating if elective surgical patients can screen themselves for being "at risk of malnutrition" in their home setting. The aim of the study was to investigate whether it is feasible for patients to answer questions related to nutritional risk screening in a home setting prior to hospital admission for surgery, and to assess the agreement between patients' perception of being at risk of malnutrition and healthcare professionals' screening, using the NRS 2002 screening tool.

Material and methods

Study design

This prospective study is part of the development and validation of Patient Safety Checklist (PASC) to be used by surgical patients before and after surgery, in a stepped wedge cluster randomized controlled trial (14) (ClinicalTrails.gov: NCT03105713).

Settings and participants

Study participants were recruited in the period between August 2019 through September 2020 from Haukeland University Hospital and Førde Central Hospital in Western Norway. Six surgical specialties were randomly drawn from eleven eligible surgical wards. All agreed to participate: Ear, Nose, Throat (ENT)-/Maxillo-Facial; Cardio-Thoracic; Neuro; Breast- and Endocrine; Gastrointestinal-; and General surgery. The study participants were selected by study personnel in cooperation with the nurses and surgeons at each ward. Inclusion criteria for the participants were: elective surgical patients aged 18 years or older, cognitively capable of answering the PASC checklist and the questions needed to fulfil NRS 2002 prior to surgery, living at home, able to give informed consent and fluent in Norwegian. Participants were prospectively recruited when scheduled to surgery by the hospital within a period of 2 to 12 weeks before surgery, depending on the patients' severity of disease and need for surgery. Patients returned their completed checklists, including nutritional risk screening questions, before discharge or by mail.

Assessment of nutritional status

According to the regional and the ~~then~~ current national guidelines (15, 16), a Norwegian translated version of NRS 2002 (17) embedded in the electronic patient record was used by healthcare professionals at the hospitals, mainly nurses, to determine whether the patients were "at risk of malnutrition" or not. This screening tool is based on four introductory

questions that detect low body mass index (BMI) (< 20.5 kg/m^2), recent weight loss, recently reduced food intake and critical illness (5). If one or more of these four questions are answered with “yes”, the patient enters the final screening. The final screening gives a total score from 0 to 7 based on more in-depth questions regarding the patient’s nutritional status (score 0 – 3) and the severity of the patient’s disease in light of nutritional requirements (score 0 – 3). In addition, patients over 70 years are given the score 1. A total score of ≥ 3 in the final screening identifies patients as being “at risk of malnutrition” (3).

In this study, the initial question regarding critical illness and the final screening questions that quantify the severity of their disease to be moderate or severe were not included, since patients in such cases would not be at home, but hospitalized. Based on the patients’ answers, study personnel calculated the NRS 2002 result after receiving the screening form back, and categorized the patients to be “at risk of malnutrition” or not. Self-registration of the nutritional information as part of the PASC (14), prior to hospitalization, is hereby called the “self-reported screening tool”. In addition, the patients were asked on the same paper form if they thought they were “at risk of malnutrition” (“yes”/”no”). These results were compared with the healthcare professionals’ nutritional risk screening results. The screening process is illustrated in **Figure 1**.

Figure 1: Illustration of the screening process in the present study. PASC: Patient Safety Checklist trial (ClinicalTrails.gov: NCT03105713)

Statistics

Descriptive analyses were conducted for the total study sample and for the subsamples attributed to different surgical wards. Summary measures for continuous variables are reported as means (SD), and categorical variables are reported as counts (percentages). The statistical package IBM SPSS Statistics v.26 was applied for descriptive analysis. To analyse the interrater reliability between the patients' and the healthcare professionals' results of the nutritional risk screening, the Kappa Measure of Agreement (κ) was investigated by using DAG_Stat (18). The associated prevalence and bias indexes (19), and degree of positive and negative agreement were also calculated. The grading system of Landis and Koch was used to describe the agreement (< 0.00, *poor*; 0.00 - 0.20, *slight*; 0.21 - 0.40, *fair*; 0.41 - 0.60, *moderate*; 0.61 - 0.80, *substantial*; 0.80 - 1.00, *almost perfect*) (20).

Ethics

The study was approved by the Regional Committee for Medical and Health Research Ethics prior to study start (Reference: 2016/1102) and the hospital managers. All study participants signed a written consent after receiving information regarding the study. Participation was voluntary and patients could withdraw at any time without consequences. Research data were handled according to the hospital's research guidelines (eProtocol: 1218-1218) and data were stored on a designated and secure research server in one of the hospitals.

Results

General characteristics

Out of 428 patients asked to participate, 215 patients (50.2 %) consented and used and returned the surgical safety checklist where the self-reported screening tool was included (**Figure 2**). Of these, 100 (46.5 %) were male, and the mean (SD) age was 57.8 (13.0) years. General characteristics of the study participants and distribution among the different surgical wards are described in **Table 1**.

Figure 2: Flowchart of the study population.

Screening performance

At home and using the self-reported screening tool, 165 (76.7 %) patients completed enough of the questions to be classified as being "at risk of malnutrition" or not. Of these, 69 (32.1%) were not screened by a healthcare professional at hospital. In total, 205 patients (95.3 %) answered "yes" or "no" to the question regarding whether they thought they were "at risk of malnutrition" prior to surgery. For the initial questions regarding the patients' BMI, weight loss and dietary intake in NRS 2002 in the self-reported screening tool, the response rates were 92.1 %, 77.7 % and 95.3 %, respectively.

At the hospital, healthcare professionals completely screened 123 patients (57.2 %) for being "at risk of malnutrition" or not, of which 96 patients (44.7 %) were screened preoperatively. Twenty-seven (12.6%) of these patients did not complete the self-screening. The healthcare professionals' response rates for the initial questions regarding the patients' BMI, weight loss and dietary intake in NRS 2002 were 59.5 %, 59.1 % and 59.5 %, respectively.

"At risk of malnutrition"

According to the answers given in the self-reported screening tool preoperatively, 11 patients (5.1 %) met the criteria for being "at risk of malnutrition" and nine patients (4.2 %) believed they were in this category. At the hospital, four of the 215 patients (1.9 %) were identified to be "at risk of malnutrition" by the healthcare professionals' screening. All these patients were screened after surgery.

Agreement regarding the initial NRS 2002 questions

The degrees of agreement between the answers of patients and healthcare professionals to the initial NRS 2002 questions were as follows: low BMI: *almost perfect* (κ = 1.00 (95% CI: 1.00, 1.00)), weight loss: *moderate* (κ = 0.51 (95% CI: 0.30, 0.72)), and reduced food intake: *slight* (κ = 0.09 (95% CI: - 0.01, 0.03)) (**Table 2**). The positive degrees of agreement for the same questions were also *almost perfect*, *moderate* and *slight*, respectively, whereas the negative degrees of agreement were *almost perfect* for them all (Table 2). The prevalence indexes were 0.51, 0.30 and 0.42 for the questions regarding low BMI, weight loss and reduced food intake, respectively, whereas the bias indexes were 0.00, 0.04 and 0.04 respectively.

Agreement regarding being "at risk of malnutrition" or not

Comparing patients identified to be "at risk of malnutrition" by healthcare professionals with the self-reported screening tool and the patients' belief regarding their own nutritional status, the degree of agreement was demonstrated to be *poor* for them both (κ = - 0.04 (95 % CI: - 0.07, 0.01) and κ = - 0.03 (95 % CI: - 0.05, -0.01), respectively) (**Table 3**). For both comparisons, there was a *poor* positive agreement, whereas the negative agreement was *almost perfect* (Table 3). Moreover, the prevalence indexes were 0.41 and 0.51, respectively, and the bias indexes were 0.01 and 0.005, respectively, for both comparisons.

The agreement between the self-reported screening tool, and the patients' belief regarding being "at risk of malnutrition" was *fair* ($\kappa = 0.27$ (95 % CI: -0.01, 0.55)) (**Table 4**). Here, the positive agreement was *fair* ($\kappa = 0.32$ (95 % CI: 0.04, 0.59)) and the negative agreement was *almost perfect* ($\kappa = 0.96$ (95 % CI: 0.93, 0.98)). The prevalence index was 0.67, and the bias index was 0.01.

Table 1: Distribution of patient characteristics and screening performance of the total study sample and subsamples

		**Haukeland University Hospital**					**Førde Central Hospital**
	Total<br>n = 215	Gastrointestinal<br>n = 37	Breast and endocrine<br>n = 45	ENT/ Maxillo-Facial<br>n = 43	Neuro<br>n = 32	Cardio-Thoracic<br>n = 36	General<br>n = 22
Age,[1] year	57.8 (13.0)	59.0 (12.8)	59.8 (9.8)	50.0 (15.7)	54.5 (9.6)	62.7 (10.0)	64.1 (14.7)
Male[2]	100 (46.5)	18 (48.6)	2 (4.4)	21 (48.8)	15 (46.9)	30 (83.3)	15 (63.6)
At hospital[2]							
Complete screening	123 (57.2)	27 (73.0)	1 (2.2)	22 (51.2)	31 (96.9)	33 (94.3)	8 (36.4)
"At risk of malnutrition"	4 (1.9)	4 (10.8)	0	0	0	0	0
At home[2]							
Complete self-screening	164 (76.7)	32 (86.5)	31 (68.9)	36 (83.7)	23 (71.9)	25 (71.4)	17 (77.3)
"At risk of malnutrition"	11 (5.1)	3 (8.1)	1 (2.2)	3 (7.0)	0	1 (2.8)	3 (13.6)
Believe to be "at risk of malnutrition"	9 (4.2)	3 (8.1)	3 (6.7)	1 (2.3)	0	0	2 (9.1)

"At risk of malnutrition" defined by Nutritional Risk Screening 2002 (5).

ENT: Ear, Nose, Throat

[1] mean (SD)

[2] n (%)

Table 2: Degrees of agreement regarding the initial NRS 2002 questions (5) between patients and healthcare professionals

	Initial NRS 2002 questions	Healthcare professionals				Positive agreement (95% CI)	Negative agreement (95% CI)	Prevalence index	Bias index	Kappa (95% CI)
	**BMI < 20.5 kg/m^2?**	*Yes*	*No*	*Missing*	*Total*					
**Patients**	*Yes*	4	0	7	11	1.00 (1.00, 1.00)	1.00 (1.00, 1.00)	0.51	0.00	1.00 (1.00, 1.00)
	No	0	113	73	186					
	Missing	0	11	7	18					
	Total	4	124	87	215					
		**Healthcare professionals**								
	**Weight loss last three months?**	*Yes*	*No*	*Missing*	*Total*	0.60 (0.41, 0.78)	0.91 (0.87, 0.96)	0.30	0.04	0.51 (0.30, 0.72)
**Patients**	*Yes*	11	12	25	48					
	No	3	76	40	119					
	Missing	0	25	23	48					
	Total	14	113	88	215					
		**Healthcare professionals**								
	**Reduced food intake last week?**	*Yes*	*No*	*Missing*	*Total*	0.19 (0.01, 0.38)	0.88 (0.84, 0.93)	0.42	0.04	0.09 (-0.01, 0.03)
**Patients**	*Yes*	*3*	*17*	16	36					
	No	8	94	67	169					
	Missing	0	6	4	10					
	Total	11	117	87	215					

NRS 2002: Nutritional Risk Screening 2002

Table 3: Degrees of agreement regarding being "at risk of malnutrition" between the patients and healthcare professionals

"At risk of malnutrition"		**"At risk of malnutrition"** *screened by healthcare professionals*				**Positive agreement (95% CI)**	**Negative agreement (95% CI)**	**Prevalence index**	**Bias index**	**Kappa (95% CI)**
		Yes	*No*	*Missing*	*Total*					
**Patient's self-screening**	*Yes*	0	5	6	11	0.00 (0.00, 0.00)	0.96 (0.93, 0.99)	0.41	0.01	-0.04 (-0.07, -0.01)
	No	3	88	63	154					
	Missing	1	26	23	50					
	Total	4	119	92	215					
		**"At risk of malnutrition"** *screened by healthcare professionals*								
		Yes	*No*	*Missing*	*Total*	0.00 (0.00, 0.00)	0.97 (0.95, 0.99)	0.51	0.005	-0.03 (-0.05, -0.01)
**Patient's belief**	*Yes*	0	3	6	9					
	No	4	109	83	196					
	Missing	0	7	3	10					
	Total	4	119	92	215					

"At risk of malnutrition" defined by Nutritional Risk Screening 2002 (NRS 2002) (5). Patient's self-screening based on answers given to NRS 2002 questions in paper form prior to admission.

Table 4: Degrees of agreement between patients' self-reported screening and their belief regarding being "at risk of malnutrition"

		**Patients believe they are "at risk of malnutrition"**				**Positive agreement (95% CI)**	**Negative agreement (95% CI)**	**Prevalence index**	**Bias index**	**Kappa (95% CI)**
		Yes	*No*	*Missing*	*Total*	0.312 (0.04, 0.59)	0.96 (0.93, 0.98)	0.67	0.01	0.27 (-0.01, 0.55)
**Self-screened to be "at risk of malnutrition"**	*Yes*	3	8	0	11					
	No	5	147	2	154					
	Missing	1	41	8	50					
	Total	9	196	10	215					

Patient's self-screening based on answers given to Nutritional Risk Screening 2002 (5) in paper form prior to admission.

Discussion

In this study, we found that most patients who met the criteria for being “at risk of malnutrition” in the self-screening tool and those who believed they were “at risk of malnutrition” preoperatively, were not identified to be so by the healthcare professionals at hospital. In most cases, this was due to missing screenings at hospital, and not necessarily due to disagreement between the patients and the healthcare professionals.

The problem of missing screening for “at risk of malnutrition” is a well-known problem both nationally and internationally (21, 22). When patients are not identified as being “at risk of malnutrition”, it is difficult to further prevent and treat the disease-related malnutrition. In this way, the chance of reducing in-hospital mortality and readmissions decreases (23). The current study demonstrated that patients tended to complete the questions needed to fulfil NRS 2002 in a home setting more often (76.7 %) than what was obtained by healthcare professionals during hospitalization (57.2 %). This is an important finding, since it illustrates a willingness and ability to perform nutritional self-screening in the patient group, which potentially could result in an increased rate of nutritional screening. User involvement has also previously been recognized as an important factor for successful patient treatment, and healthcare professionals are encouraged to increase user involvement in all aspects of their work, both within clinical work and research (8, 24).

Early intervention in vulnerable groups to prevent malnutrition was voted as the most prioritized research question within the field of adult malnutrition and nutritional screening in healthcare by a partnership between patients, careers and healthcare professionals (25). The underlying cause of surgery, as well as fear of surgery and/or pain or illness may decrease patients’ appetite and lead to reduced dietary intake and weight loss preoperatively. This may further lead to a suboptimal nutritional status prior to surgery, and thus increase the risk of

postoperative complications (7). To be able to treat patients who are “at risk of malnutrition” prior to surgery, early screening has to be conducted. Here, an organizational challenge occurs, since the patient responsibility lies between the primary and secondary healthcare system in the preoperative phase, and the responsibility for nutritional screening and treatment may become unclear. Thus, questions regarding risk of malnutrition are an important part of the patients’ checklist prior to surgery.

For hospitals, the Norwegian guidelines for preventing and treating malnutrition recommend patients be screened for being “at risk of malnutrition” within 24 hours after admission (4). Of note, the organization of healthcare services in Norway has undergone some major changes during the last two decades, which has led to a reduced length of stay at hospital. As a result, many patients, who previously could been screened for being “at risk of malnutrition” at hospital within 24 hours, are now discharged. This leads to a lower chance of preventing malnutrition, as the patients “at risk of malnutrition” will not be identified, and thereby treated, during their hospital stay. The low prevalence of patients staying overnight at the ward for Breast and Endocrine surgery may explain why this ward has such a low screening performance (2.2 %) among the patients included in the current study (see Table 1). Moreover, a significant number of these patients are having surgery for breast cancer. Since this patient group is known to have problems related to unintended weight gain in the postoperative phase (26), the focus on malnutrition may be decreased. Notably, 6.7 % of the study participants at this ward thought they were “at risk of malnutrition”, but were not screened for this during hospitalization.

The degree of agreement between patients who identified themselves as being “at risk of malnutrition” by the self-reporting screening tool and by personal belief, with the result from the screening at the hospital, was *poor*. This was partly due to high prevalence indexes, since the proportion of agreements on the positive classification (*poor*) differed from that of the

negative classification (*almost perfect*). Of note, more patients were not screened for being "at risk of malnutrition" at hospital (missing data) than there were cases of disagreement in the screening results.

None of the patients who were identified at hospital to be "at risk of malnutrition" by the healthcare professionals were identified to be so by the criteria in the self-reporting screening tool, and none of them thought they were so prior to admission. Of note, these patients were only screened by the healthcare professionals after the surgery. Thus, the *poor* degree of agreement may be caused by the time difference, since we know that surgery and hospitalization may lead to loss of appetite, and reduced dietary intake and body weight (7). Notably, this illustrates the importance of optimal nutrition reserves prior to surgery, and at least weekly screening routines at the hospital (27), since the nutritional status may worsen in a short time.

In an attempt to overcome the barriers for low screening performance for "at risk of malnutrition", the Norwegian Directorate of Health recently published new guidelines for preventing and treating malnutrition (4). In these revised guidelines, there was a strong consensus towards implementing MST (28) as a new screening tool for both the primary and secondary healthcare system. MST consist of two questions: one regarding weight changes and one regarding changes in dietary intake, and is therefore easier to use than NRS 2002. Since the current study demonstrated patients were willing to screen themselves prior to elective surgery, and a previous study has reported patients successfully screened themselves with MST in an outpatient setting (13), there is reason to believe that MST can also be used in a home setting for self-screening. Of note, if such, it is important to facilitate a treatment offer for those who identify themselves as being "at risk of malnutrition".

Strengths and limitations

The strength of this study is the prospective design and the use of the daily nutritional risk screening routines at six different surgical wards from two hospitals. The main limitation is the missing number of patients being screened for being “at risk of malnutrition” at hospital, since this decreases the possibility of comparing the screening results between patients and healthcare professionals. Moreover, there was a high number of non-responder patients. Previously published analysis showed no difference between genders on responding, but demonstrated that the central community hospital had a higher number of non-responders, possible indicating that patients with more complex surgery and medical conditions were the ones who participated in the study (14). In addition, the modifications to NRS 2002 to customize the screening tool for patients’ self-screening may have affected its reliability. Of note, similar modifications have also been suggested to self-reporting screening by using MUST (10). Further limitations of the study include that healthcare professionals most often screened the patients after surgery, whereas the patients screened themselves prior to surgery, and that the response rate may induce a selection bias.

Clinical relevance

This study demonstrated a potential whereby elective patients can answer questions used to assess their nutritional status prior to surgery in a home setting. If this is further developed, there is a possibility to identify those patients who are “at risk of malnutrition” prior to hospitalization and utilize the waiting time prior to surgery to safeguard and improve their nutritional status. Further investigation should include the electronic formats that may improve the accuracy and ease of use of self-screening, and include an associated treatment offer for those who need such.

Conclusion

Preoperative self-screening in a home setting may be a feasible method to increase the number of elective surgical patients screened for being “at risk of malnutrition”. Of note, this may not be valid for all elective surgical patients, and should therefore be viewed as a supplement and not a replacement for healthcare professionals’ screening.

Acknowledgements

We would like to express our thanks to all the patients who participated in the study and to the staff at the participating surgical wards for practical help during patient recruitment.

References

1. Weimann A, Braga M, Carli F, Higashiguchi T, Hubner M, Klek S, et al. ESPEN guideline: Clinical nutrition in surgery. Clin Nutr. 2017;36(3):623-50.
2. World Health Organization. WHO Guidelines for Safe Surgery 2009 [Available from: http://apps.who.int/iris/bitstream/handle/10665/44185/9789241598552_eng.pdf?sequence=1.
3. Kondrup J, Allison SP, Elia M, Vellas B, Plauth M, Educational, et al. ESPEN guidelines for nutrition screening 2002. Clin Nutr. 2003;22(4):415-21.
4. The Norwegian Directorate of Health. Preventing and Treatment of Malnutrition 2021 [Available from: https://www.helsedirektoratet.no/retningslinjer/forebygging-og-behandling-av-underernaering.
5. Kondrup J, Rasmussen HH, Hamberg O, Stanga Z, Ad Hoc EWG. Nutritional risk screening (NRS 2002): a new method based on an analysis of controlled clinical trials. Clin Nutr. 2003;22(3):321-36.
6. Lobo DN, Gianotti L, Adiamah A, Barazzoni R, Deutz NEP, Dhatariya K, et al. Perioperative nutrition: Recommendations from the ESPEN expert group. Clin Nutr. 2020;39(11):3211-27.
7. Gillis C, Wischmeyer PE. Pre-operative nutrition and the elective surgical patient: why, how and what? Anaesthesia. 2019;74 Suppl 1:27-35.
8. World Health Organization. Exploring patient participation in reducing health-care-related safety risks: Regional Office for Europe; 2013 [Available from: https://apps.who.int/iris/handle/10665/326442.
9. Forum. EP. Briefing Paper on Oatient Safety with a focus on the role of patients and families 2016 [Available from: http://www.eu-patient.eu/globalassets/policy/patientssafety/patient-safety-briefing-paper.pdf.
10. Cawood AL, Elia M, Sharp SK, Stratton RJ. Malnutrition self-screening by using MUST in hospital outpatients: validity, reliability, and ease of use. Am J Clin Nutr. 2012;96(5):1000-7.
11. Harris K, Softeland E, Moi AL, Harthug S, Storesund A, Jesuthasan S, et al. Patients' and healthcare workers' recommendations for a surgical patient safety checklist - a qualitative study. BMC Health Serv Res. 2020;20(1):43.
12. Gabrielson DK, Scaffidi D, Leung E, Stoyanoff L, Robinson J, Nisenbaum R, et al. Use of an abridged scored Patient-Generated Subjective Global Assessment (abPG-SGA) as a nutritional screening tool for cancer patients in an outpatient setting. Nutr Cancer. 2013;65(2):234-9.
13. Di Bella A, Blake C, Young A, Pelecanos A, Brown T. Reliability of Patient-Led Screening with the Malnutrition Screening Tool: Agreement between Patient and Health Care Professional Scores in the Cancer Care Ambulatory Setting. J Acad Nutr Diet. 2018;118(6):1065-71.
14. Harris K, Softeland E, Moi AL, Harthug S, Ravnoy M, Storesund A, et al. Development and validation of patients' surgical safety checklist. BMC Health Serv Res. 2022;22(1):259.
15. The Norwegian Directorate of Health. National Guidelines for Preventing and Treatment of Malnutrition 2009 [Available from: https://www.helsedirektoratet.no/retningslinjer/underernaering/Forebygging%20og%20behandling%20av%20underern%C3%A6ring%20%E2%80%93%20Nasjonal%20faglig%20retningslinje.pdf/_/attachment/inline/a69263ff-b40e-4d23-a7c6-67acce28a88e:48ba11770eaa7f7a1d18145aa0e1f30086ca5dcd/Forebygging%20og%20behandling%20av%20underern%C3%A6ring%20%E2%80%93%20Nasjonal%20faglig%20retningslinje.pdf.

16. Western Norway Regional Health Authority. Case 12215 Program for patient safety in Western Norway Regional Health Authority - status as of November 2015 2015 [Available from: https://helse-vest.no/seksjon/styresaker/Documents/2015/10.12.2015/Sak%2012215%20Program%20for%20pasienttryggleik%20i%20Helse%20Vest%20-%20status%20per%20november%202015.pdf.

17. Norwegian Society for Clinical Nutrition and Metabolism. Good nutritional practice Oslo/Bergen/Trondheim/Tromsø2014 [Available from: http://www.nske.no/pdf/290517_god_ernaringspraksis.pdf.

18. Mackinnon A. A spreadsheet for the calculation of comprehensive statistics for the assessment of diagnostic tests and inter-rater agreement. . Computers in Biology and Medicine. 2000;30(3):127-34.

19. Sim J, Wright CC. The kappa statistic in reliability studies: use, interpretation, and sample size requirements. Phys Ther. 2005;85(3):257-68.

20. Landis JR, Koch GG. The measurement of observer agreement for categorical data. Biometrics. 1977;33(1):159-74.

21. Schuetz P, Fehr R, Baechli V, Geiser M, Deiss M, Gomes F, et al. Individualised nutritional support in medical inpatients at nutritional risk: a randomised clinical trial. Lancet. 2019;393(10188):2312-21.

22. Skeie E, Tangvik RJ, Nymo LS, Harthug S, Lassen K, Viste A. Weight loss and BMI criteria in GLIM's definition of malnutrition is associated with postoperative complications following abdominal resections - Results from a National Quality Registry. Clin Nutr. 2020;39(5):1593-9.

23. Kaegi-Braun N, Mueller M, Schuetz P, Mueller B, Kutz A. Evaluation of Nutritional Support and In-Hospital Mortality in Patients With Malnutrition. JAMA Netw Open. 2021;4(1):e2033433.

24. Harris K, Russ S. Patient-completed safety checklists as an empowerment tool for patient involvement in patient safety: concepts, considerations and recommendations. Future Healthc J. 2021;8(3):e567-e73.

25. Jones DJ, Baldwin C, Lal S, Stanmore E, Farrer K, Connolly E, et al. Priority setting for adult malnutrition and nutritional screening in healthcare: a James Lind Alliance. J Hum Nutr Diet. 2020;33(2):274-83.

26. Irwin ML, McTiernan A, Baumgartner RN, Baumgartner KB, Bernstein L, Gilliland FD, et al. Changes in body fat and weight after a breast cancer diagnosis: influence of demographic, prognostic, and lifestyle factors. J Clin Oncol. 2005;23(4):774-82.

27. The Norwegian Directorate of Health. Package of measures for the prevention and treatment of malnutrition (hospital) 2018 [Available from: https://pasientsikkerhetsprogrammet.no/om-oss/innsatsomrader/ernaering/_/attachment/inline/80302130-98eb-4c67-a439-293bf4856066:d0438f00f6268ebd5a68954b07e82d090ef9968f/tiltakspakke-for-forebygging-og-behandling-av-underernaering-i-sykehus.pdf.

28. Ferguson M, Capra S, Bauer J, Banks M. Development of a valid and reliable malnutrition screening tool for adult acute hospital patients. Nutrition. 1999;15(6):458-64.

Figure 1

Click here to access/download;Figure;Figure 1.pdf

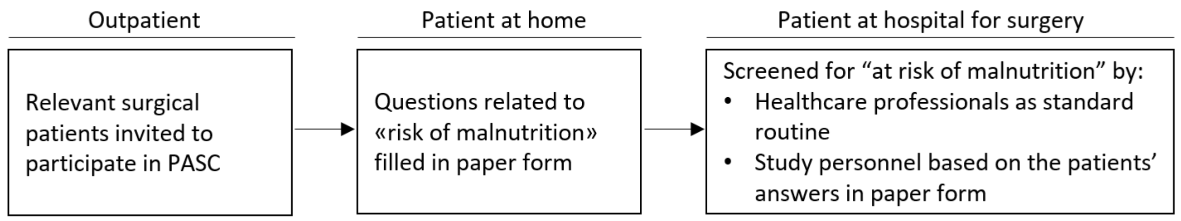

Figure 2

Click here to access/download;Figure;Figure 2.pdf

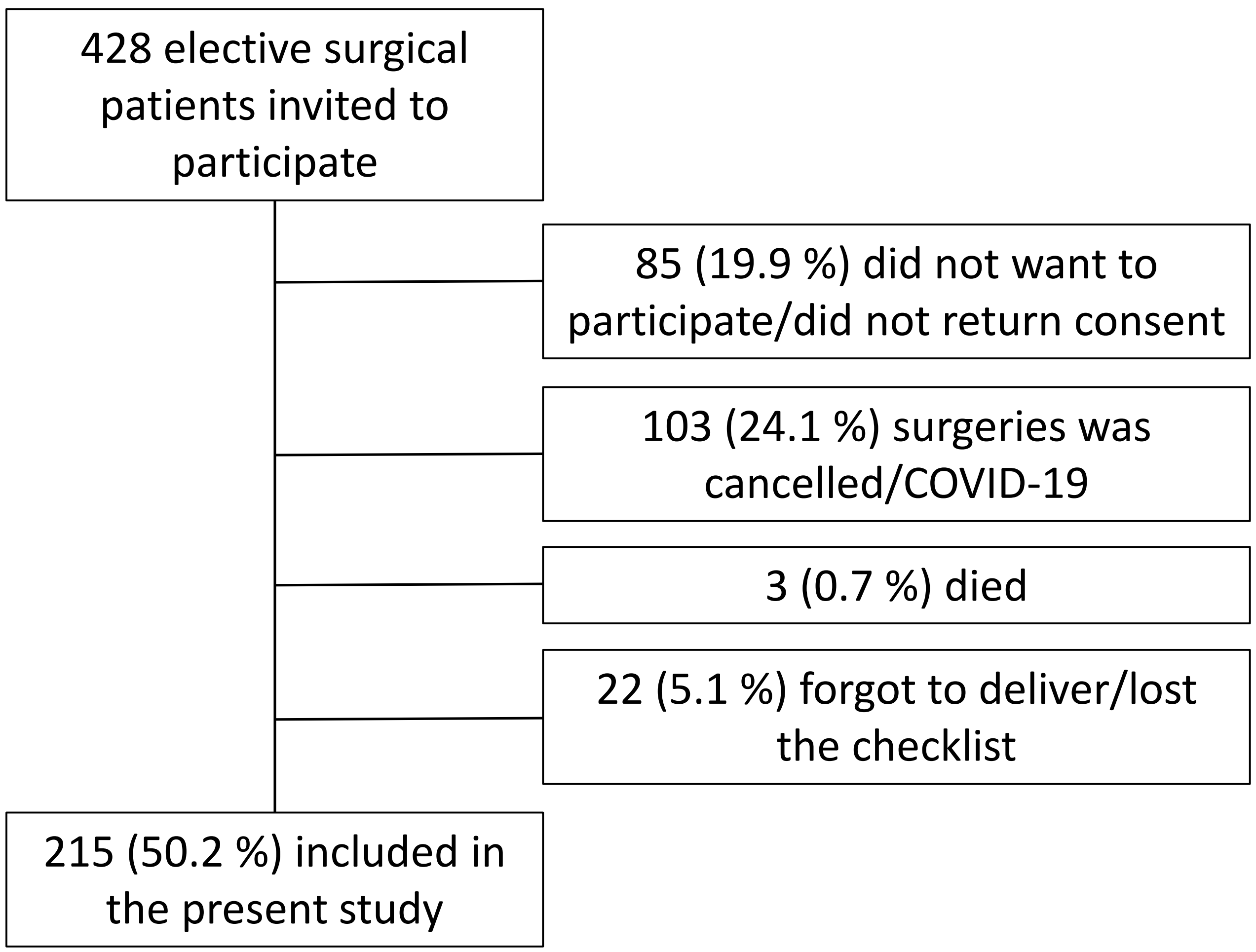

Data material

Click here to access/download

Supporting Information

AdditionalFile.pdf

Click here to access/download
Supporting Information
CONSORT extension for Pilot and Feasibility Trials Checklist.doc

Click here to access/download
Supporting Information
Study protocol_ClinicalTrails.gov.pdf
