# Supplementary material for: Is self-screening for 'at risk of malnutrition' feasible in a home setting?
Source: PLoS One. 2024 Apr 16;19(4):e0299305. doi: 10.1371/journal.pone.0299305 (PMC11020964; doi:10.1371/journal.pone.0299305)
Supplement: Attachment — Submitted filename: Respons to the editors and reviewers comments..docx [file pone.0299305.s004.docx]

Maret G Traber, PhD	Bergen, April 16^th^ 2023

Academic Editor

PLOS ONE

Dear Editor and Reviewer,

I hereby submit the revised version of our manuscript “Practicability of self-screening for “at risk of malnutrition” among surgical patients’ in a home setting” (PONE-D-22-30022).

We thank the Editor and Reviewer for a thorough and thoughtful evaluation of the manuscript, as well as for critical and helpful comments. In our revision of the manuscript, we have addressed all the objections and suggestions raised to the best of our ability.

Regarding the Competing Interests section, we state as requested by the Journal Requirements the following: “This does not alter our adherence to PLOS ONE policies on sharing data and materials.”. The Journal Requirements also requested captions for our Supporting Information files which can now be found in the end of the manuscript. One of these also include an anonymized dataset.

Below, you will find the point-by-point response to the Editor and Reviewer. All lines and pages referred to apply to the Revised Manuscript with Track Changes. We hope that our revised version has addressed their comments satisfactorily and is now at a level that it can be deemed acceptable for publication in PLOS ONE.

We look forward to hear from you.

Best regards,

Eli Skeie

Corresponding Author

**Response to the Editor’s Comments**

**#1** The reviewer has highlighted a critical area of confusion concerning the word “feasibility”. Authors frequently are asked in clinical trials to state the kind of study design in the title. As noted “Feasibility studies are pieces of research done before a main study to answer the question 'Can this study be done? ' They are used to estimate important parameters that are needed to design the main study.”(see: https://www.nihr.ac.uk/glossary/?letter=F&postcategory=-1). In other words, a feasibility study is one that has not collected data, but has a study design and statistical support for that design. The study the authors have carried out might be better described as a “pilot trial” that shows positive results. To avoid the difficulty with the multiple meanings of feasibility, I suggest that the authors carefully examine their manuscript and locate all instances of “feasibility”, then replace it another word that conveys their meaning, such as “practicability”.

***Author response****:* *We agree in this comment, and have now changed the word/wording of “feasibility” into “practicability”. This can be seen in the title, as well as in the aim (line 78, page 5) and in the conclusion (line 48, page 3 (abstract), and line 307, page 20).*

**#2** The other important point the reviewer makes is that they have not done a power calculation to estimate how many subjects they need to claim that the results are statistically significant. A power calculation may not be possible without the data they collected, suggesting another reason to call this a pilot trial.

***Author response****:* *In the PASC trial protocol, a power calculation was performed to calculate the numbers needed per cluster. The initial numbers were 50 patients per cluster per month. We estimated that 50 patients per cluster would be sufficient to investigate how feasible this was, however, the COVID-19 pandemic situation influence on the numbers of patients recruited. We have provided the rationale for the sample size under Material and methods section, pages 6, lines 84-87: “Based on the power calculation in the PASC trail protocol, which was performed to calculate the numbers of elective surgical patients needed per cluster in the larger SW-CRCT, a need of 300 elective patients was estimated.”*

**Response to the Reviewer’s Comments**

**Reviewer #1**: This study is presented as a feasibility study, however it doesn't seem to have actually been intended to assess feasibility of an intervention, as the objectives are presented as "investigating whether self-screening for nutritional risk factors prior to surgery in a home setting will increase the amount of patients screened for nutritional risk, and secondly, to compare their screening results with healthcare professionals..." (lines 26 to 29). Going by this, the objective of this study is to assess effectiveness of an intervention. The authors should be clearer about what the study was intending to achieve as the title, objectives in the abstract, and objectives at the end of the methods section are at odds.

If the authors did indeed aim to "investigate whether self screening... will increase the amount of patients screened...", then there are a number of aspects that need to be clearly reported, including the outcomes of the study and how they were measured, and also compared across groups. However, if the study aimed to investigate feasibility as stated in the title and in line 75, then the authors need to clearly describe what the feasibility endpoints are, ie. what outcome would they need to observe to conclude that self-screening was feasible. Additionally, the authors should describe how they determined the appropriate size of the study - a reader cannot tell whether the 215 patients who participated were sufficient to address the objectives of the study.

***Author response****: We agree in these comments, which is also commented by the Editor. Please see “Author response” to the Editor’s comments.*
