# Supplementary material for: Is self-screening for 'at risk of malnutrition' feasible in a home setting?
Source: PLoS One. 2024 Apr 16;19(4):e0299305. doi: 10.1371/journal.pone.0299305 (PMC11020964; doi:10.1371/journal.pone.0299305)
Supplement: Attachment — Submitted filename: Response to Reviewers.docx [file pone.0299305.s006.docx]

PONE-D-22-30022R1

*Is self-screening for 'at risk of malnutrition' feasible in a home setting?*

(ClinicalTrails.gov: NCT03105713)

**Response to the reviewers**

## Reviewer #1: (previous round)

*... assess whether the revision of 'feasible' to 'practicable' is a meaningful difference, and whether this study is assessing feasibility/practicability or effectiveness and reliability. Please make any revisions necessary to clarify this matter.*

Author response

This study is part of a larger feasibility study published in BMC March 2023: *Harris et al: Feasibility of implementation of a surgical patient checklist. A cross section prospective evaluation.* Hereby, we submit a paper aiming to investigate whether self-screening for nutritional risk as part of the surgical patient’s checklist (PASC) is feasible. This is an important test prior to a larger project. This is clarified in following places:

Line 1-4 (title) Is self-screening for 'at risk of malnutrition' feasible in a home setting? (ClinicalTrails.gov: NCT03105713),

Line 103-104 (method) in the 'Revised Manuscript with Track Changes': “This prospective study is part of the development and validation of Patient Safety Checklist (PASC) to be used by surgical patients before and after surgery, in a stepped wedge cluster randomized controlled trial (SW-CRCT).”

Line 402-404 (references): 14. Harris K, Søfteland E, Moi AL, Harthug S, Ravnøy M, Storesund A, Jurmy E, Skeie E, Wæhle HV, Sevdalis N, Haugen AS. [Feasibility of implementing a surgical patient safety checklist: prospective cross-sectional evaluation](https://www.ncbi.nlm.nih.gov/pmc/articles/PMC10040905/). Pilot **Feasibility** Stud. **2023**; 9: 52.


Reviewer's Responses to Questions

**Comments to the Author**
*1. If the authors have adequately addressed your comments raised in a previous round of review and you feel that this manuscript is now acceptable for publication, you may indicate that here to bypass the “Comments to the Author” section, enter your conflict of interest statement in the “Confidential to Editor” section, and submit your "Accept" recommendation.*

*Reviewer #2: All comments have been addressed.*

*Reviewer #3: (No Response)*

*Reviewer #4: (No Response)*

*Reviewer #5: (No Response)*

Author’s response

Thank you for your positive response.

*2. Is the manuscript technically sound, and do the data support the conclusions?
The manuscript must describe a technically sound piece of scientific research with data that supports the conclusions. Experiments must have been conducted rigorously, with appropriate controls, replication, and sample sizes. The conclusions must be drawn appropriately based on the data presented.*

*Reviewer #2: Yes*

*Reviewer #3: Partly*

*Reviewer #4: Partly*

*Reviewer #5: Yes*

Author’s response

Thank you for your response. This has now improved and will be clarified further in this response letter.

*3. Has the statistical analysis been performed appropriately and rigorously?*

*Reviewer #2: Yes*

*Reviewer #3: No*

*Reviewer #4: Yes*

*Reviewer #5: Yes*

Author’s response

Thank you for this remark. We agree with reviewer #3 regarding the use of Kappa Measure of Agreement and appreciate this comment. The analyses are now performed with Fleiss multi rater Kappa with overall agreement.

Line 163-167 (statistics) in the 'Revised Manuscript with Track Changes': To analyse the interrater reliability between the patients’ and the healthcare professionals’ results of the nutritional risk screening, the Fleiss multi rater Kappa with overall agreement (κ) was investigated by using SPSS version 26

Line 208-210, 219-220, 224-225 (results) in the 'Revised Manuscript with Track Changes': Revision is made in table 2, table 3 and table 4.

*4. Have the authors made all data underlying the findings in their manuscript fully available?
The* [*PLOS Data policy*](http://www.plosone.org/static/policies.action#sharing) *requires authors to make all data underlying the findings described in their manuscript fully available without restriction, with rare exception (please refer to the Data Availability Statement in the manuscript PDF file). The data should be provided as part of the manuscript or its supporting information or deposited in a public repository. For example, in addition to summary statistics, the data points behind means, medians and variance measures should be available. If there are restrictions on publicly sharing data—e.g. participant privacy or use of data from a third party—those must be specified.*

*Reviewer #2: Yes*

*Reviewer #3: Yes*

*Reviewer #4: Yes*

*Reviewer #5: Yes*

Author’s response

Thank you for your positive response.

*5. Is the manuscript presented in an intelligible fashion and written in standard English?
PLOS ONE does not copyedit accepted manuscripts, so the language in submitted articles must be clear, correct, and unambiguous. Any typographical or grammatical errors should be corrected at revision, so please note any specific errors here.*

*Reviewer #2: Yes*

*Reviewer #3: Yes*

*Reviewer #4: Yes*

*Reviewer #5: Yes*

Author’s response

Thank you for your positive response.

*6. Review Comments to the Author
Please use the space provided to explain your answers to the questions above. You may also include additional comments for the author, including concerns about dual publication, research ethics, or publication ethics. (Please upload your review as an attachment if it exceeds 20,000 characters)*

## Reviewer #2

*Generally, the content in the manuscript is well described.
Please make a minor technical amendment to the manuscript before finalized submission. Insert the comment in the attached file.*

*Comments from the attached file*

1. *Line 90: How about participants/health care professionals from the eligible surgical wards?*
2. *Line 127: Remove “then” in the sentence “According to the regional and the then current national guidelines.”*
3. *Line 308: Note that health care professionals are well trained for nutritional screening.*

Authors response

Thank you for these comments.

1. The health care professionals were working at the recruited surgical wards and performed nutritional risk screening routinely as usual, according to hospitals guidelines.

2. Line 127 is now revised. The new sentence is “According to the regional and the current national guidelines”.

3. This important point is now included in the revised discussion:

Line 278 : For hospitals, the Norwegian guidelines for preventing and treating malnutrition recommend patients be screened for being “at risk of malnutrition” within 24 hours after admission (4). Self-screening at home will not change the hospitals responsibility for following these guidelines. The health care personnel are trained for screening and responsible for taking care of the risk patients by assessment, nutritional treatment and finally to communicate information regarding nutritional therapy to next level of care.

## Reviewer #3

*This manuscript aims to investigate the important question of how we can improve access to nutrition screening preoperatively to identify patients at risk of malnutrition and therefore at increased risk of postoperative complications. The manuscript contains some interesting data but needs major revisions to enable interpretation of the findings.*

Authors response

Thank you for your comment. The manuscript has been revised according to alle the comments from the reviewers and the interpretation of the findings is improved. This is described in detail in this response letter.

*Abstract*

1. *Line 28 - amount should be number of patients.*
2. *Line 45 - how many patients had both a healthcare professional and a self-screening completed? This is the most important number and should be reported.*
3. *What was the time difference between the screening tools being completed?*
4. *Line 47 - 48 - the conclusion does not reflect the results section. The agreement is stated as "poor".*

Authors response

1. “Amount of patients” is now changed to “*number* of patients” (line 48).
2. The number of patients who had both a healthcare professional and a self-screening completed is 96 (67.9 %), this is pointed out in the results.

Line 189-190: *Of these, 96 (67.9%) were screened by a healthcare professional at hospital.*

3. The self-screening was performed from 12 to two weeks before surgery. The health care workers screened the patients from two weeks and until the day before surgery. This variation depends on each surgical ward’s routine for the surgical admission and in some cases the screening was fulfilled after surgery. This is pointed out in the abstract and in the method section.

Line 50-55 (abstract): As part of the checklist, patients were preoperatively in a home-setting, until 3 months prior to surgery asked questions needed to identify “at risk of malnutrition” by a moderated version of the Nutritional Risk Screening tool (NRS 2002). The original screening tool (NRS 2002) was used as a standard routine to identify “at risk of malnutrition” by healthcare professionals at hospital, from two week until the day prior to surgery.

Line 155 (method): Self-registration of the nutritional information as part of the PASC (14), “*at home”*, is hereby called the “self-reported screening tool” and was performed up to 3 months prior to surgery.

Line 158-160 (method): These results were compared with the healthcare professionals’ nutritional risk screening results which was performed at the hospital 2 weeks before and until the day before surgery and in some cases after surgery.

4. The abstract conclusion is now revised and in line with the results.

Line 68: Preoperatively self-screening in a home setting may be a feasible method to increase the number of elective surgical patients screened for risk of malnutrition. The patients and healthcare workers agreement regarding low body mass index, weight loss and reduced food intake was *almost perfect*, *moderate,* and *slight*, respectively. However, the over-all agreement on NRS2002 showed that agreement regarding classification of “At risk of malnutrition” was *poor.* Hence, NRS 2002 may not be a recommended tool for nutritional risk self-screening for surgical patients at home.

*Methods*

1. *Line 87 - how was this sample size calculated? Please include further details.*
2. *Line 96 - 98 - were patients recruited while an inpatient or at an outpatient clinic appointment? It is not clear. Why did health professionals not complete the nutrition screening at this point in time rather than when patients were admitted for surgery?*
3. *Please add further details about the NRS 2002 questions and the timing of screening.*
4. *Lines 105 to 107 - how did calculate BMI? Was this based on self-reported weight and height? Did they have to use tables on paper to work this out? Please include the exact screening tool questions that were used in this study in a table or figure or supplementary information.*
5. *Lines 113- 117. It is unclear if the scoring system for "at risk of malnutrition" was changed given patients did not complete all questions. Please include a comment about whether this is an acceptable use of the screening tool.*
6. *Section starting Line 101. When did patients complete the tool? If a patient completes the tool 12 weeks prior to the health professional completing the tool the inter-rater agreement cannot be compared b/c they are not assessing the same state. It is likely that changes in weight and food intake will occur during this period.*
7. *Line 119 - 120 - has this question (... the patients were asked on the same paper form if they thought they were “at risk of malnutrition” (“yes”/”no”)) been used in previous studies? Please provide evidence to support its inclusion or at least justify why it has been included.*

Authors response

1. In our revised manuscript it is clearer that our study was part of a larger project, and the power calculating was performed for this larger study. We have changed the paper in ref 14 to clarify this.

Line 112 (methods): The power calculation for this study was based on the PASC trail protocol. It was calculated a need of a total of 300, elective surgical patients, across six clusters over 14 months (14).

2. Participants were recruited by study personnel or health care workers after receiving the date for surgery, either at the outpatient clinic appointment or by mail. The health care personnel performed the screening according to the hospital’s routines which is now explained in the manuscript:

Line 120 (methods): The study participants were recruited by study personnel in cooperation with the nurses and surgeons at each ward. This was done either at the outpatient clinic appointment or by letter in the mail, after the patient had received a date for surgery.

Line 151 (methods): ... the healthcare professionals’ nutritional risk screening results which was performed at the hospital 2 weeks before and until the day before surgery and in some cases after surgery.

3. See authors response in the abstract section, number 3 (over).
4. Thank you for the opportunity to clarify this. We have re-vised Figure 1, showing both the screening procedure and the exact self-screening tool, which is the nutrition questions from NRS2002 included in the patient surgery checklist. Based on patients answering the questions in Figure 1, study personnel calculated BMI and the NRS2002-scores. This is explained in line 142-149.

Line 142-149 (methods): NRS 2002 is designed for use by healthcare personnel. Some adjustments were made for patients’ self-screening with NRS2002; First, the initial question regarding critical illness and the final screening questions that quantify the severity of their disease to be moderate or severe were left out, since patients in such cases would not be at home but hospitalized. Secondly, the screening tool was re-design to be more patient user-friendly, see Figure 1. Finally, based on the patients’ answers to the questions and their age, study personnel calculated the BMI and, NRS 2002 scores, and categorized the patients to be “at risk of malnutrition” (≥ 3 scores) or not (< 3 scores).

5. The modification of NRS 2002 is discussed as a weakness of the study:

Line 342 (strengths and limitations): In addition, the modifications to NRS 2002 to customize the screening tool for patients’ self-screening may have affected its reliability. Of note, similar modifications have also been suggested to self-reporting screening by using MUST (10).

6. See authors response to the abstract section number 3 (over). We cannot exclude the possibility of bias due to this timeline, as discussed as a weakness of the study in line 345-347.

Line 345-347 (strengths and limitations): Further limitations of the study include that healthcare professionals most often screened the patients after surgery, whereas the patients screened themselves prior to surgery, and that the response rate may induce a selection bias.

7. The question whether the patient thought they were “at risk of malnutrition” is a question in the surgical patient’s checklist (PASC) and not a part of the screening tool NRS 2002. We are not aware of others using this question. We think the patient’s perception regarding their nutritional status is of interest, even this single question is of minor value. We have some qualitative data analyses in progress to explore this.

*Statistical methods
This section does not detail which kappa measure of agreement was used. Please review accordingly. Assumption 5 of Cohen's kappa was not met - "Assumption #5: The same two raters are used to judge all observations (e.g., patients). This has been referred to as having fixed or unique raters. If different raters were used for each observation (e.g., patient), Cohen's kappa is not the appropriate test to use. However, in this latter case, you could use Fleiss' kappa instead, which allows randomly chosen raters for each observation (e.g., patient)."*

Authors response

Thank you for this comment. We agree with the reviewer and have performed a new analysis by Fleiss multi rater Kappa with overall agreement. This method assess the [reliability of agreement](https://en.wikipedia.org/wiki/Inter-rater_reliability) between a fixed number of [raters](https://en.wikipedia.org/wiki/Rater) when assigning [categorical ratings](https://en.wikipedia.org/wiki/Categorical_rating) to a number of items or classifying items (Wiki). This is changed in the result section, in Table 2, 3 and 4, and outlined in the statistics section. Further, we had removed the analyses of positive degree of agreement, negative degree of agreement, the prevalence index, and the bias index.

*Results*

1. *Line 169 - were the patients who believed they were at risk of malnutrition the same patients who were identified as at risk of malnutrition on the NRS2002? This is important to know.*
2. *Line 171-172 "All these patients were screened after surgery". The use of the screening tool after surgery is not detailed in the methods section. Please make it clear in the. methods section when HPs and patients completed the screening tool.*
3. *Line 192 - be aware of over interpreting the statistics. The number of patients identified at risk of malnutrition was only 5% of the population and the agreement statistic is based on a small sample of 9 versus 11 screening tool results.*

Authors response

1. This important result is presented in detail in the following section in the manuscripts: Agreement regarding being “at risk of malnutrition” or not (Line 222-231). The agreement between being and believe being “at risk of malnutrition” or not is outlined in the results line 230-231 and visualized in detail in Table 4.

Line 230: The agreement between the self-reported screening tool, and the patients’ belief regarding being “at risk of malnutrition” was fair (κ = 0.27 (95 % CI: -0.12, 0.30)) (Table 4).

2. Thank you for this comment. In cases where the routine screening failed (n=27), the patients were screened after surgery. This is now clarified in the manuscript.

Line 153 (methods): … the healthcare professionals’ nutritional risk screening results which was performed at the hospital 2 weeks before and until the day before surgery and in some cases after surgery.

3. Thank you for this important remark. This study was performed to get experience with the PASC checklist and nutrition risk self-screening. Based on our experiences, we cannot recommend the NRS2002 have chosen another screening tool for the main project, and we will recommend other to do the same, even the sample size is small.

*Discussion*

1. *Line 230 - this needs a reference please.*
2. *Line 255 - it is not clear what the "high prevalence indices" is referring to.*
3. *Line 260 - 262 - these results are not clearly presented in the results section. Please revise accordingly.*
4. *Line 264 - please add further details about the time difference to the results section. This is very important as timing of risk of malnutrition prior to surgery dictates the type of intervention that could be provided to reduce risk preoperatively.*
5. *Line 278 to 279 - yes very important point!*
6. *Line 284 - the time delay here is also important. For example, screening by health professionals at the time of booking surgery date would be a more accurate method to compare with self-screening at time of booking.*

Authors response

1. We do not have a reference to this sentence and has therefor revised it:

Line 277 (discussion): Several factors may affect the patient’s appetite before surgery, such as the underlying cause of surgery, as well as fear of surgery and/or pain or illness.

2. We agree about the prevalence index and have deleted this from the text and tables.
3. We think this is clearly presented in Table 2, however vi understand that “… the criteria in the self-reporting screening tool, “might be unclear and have changed this.

Line 314 (discussion): “… the questions in the self-reporting screening tool, …

4. See authors response to **Reviewer #3 the abstract section number 3 (over)**.
5. Thank you.
6. Yes, indeed we agree. Hopefully, patients will routinely be screened for malnutrition at the time of booking surgery date in the future. And hopefully might this paper contribute to increased attention to this possibility.

## *Reviewer #4:*

## *I reviewed the article "Practicability of self-screening for "at risk of malnutrition" among surgical patients in a home setting.” The study's primary purpose is to investigate and compare the agreement rates between preoperatively measured self-reported malnutrition screening at home by patients and at the hospital by nurses. Although the study has a good design and presentation, some major methodological points make the study lower quality.*

## 1. *Missing data when choosing the patients in the study is much more than expected, which may cause a bias in the analyses.*

## 2. *The hypothesis and purpose of the study needed to be well documented in the paper. I could not understand what this work would add to this area. Preoperatively self-measured malnutrition rate should be like measurements in the hospital. However, kappa analysis results show shallow coefficient scores.*

## Authors response

1. We have received a lot of valuable comments that have been very helpful to improve the manuscript significantly. We agree that the high amount of missing data may cause a bias, as expressed in the strengths and limitations section (Line 333-347).
2. The aim of this study is now more clearly written line 101-106. We do agree that the patients preoperatively self-measured malnutrition rate should be similar to measurements performed by health care personnel in the hospital. Therefore, our conclusion is that NRS 2002 may not be the preferred tool for self-screening at home.

Line 357 (conclusion): Completed by three out of four patients, preoperative self-screening in a home setting may be a feasible method to increase the number of elective surgical patients screened for being “at risk of malnutrition” before surgery. … Even high agreement between parts of the NRS 2002, the agreement regarding the final categorisation of being at risk for malnutrition or not was poor. Hence, we recommend exploring other self-screening tool to identify nutritional risk in a home setting.

## Reviewer #5:

*ABSTRACT*

1. *Results – might be useful to include the numbers screened in hospital prior to surgery. I got the impression that those screened pre surgery was more important than post.*
2. *Conclusions – might also be useful to mention here that hospital screening for malnutrition risk doesn’t always occur before surgery, so limiting opportunity to treat and better prepare for surgery.*

Authors response

1. We have compared the patients self-screening with screening performed by health care personnel at hospital. We have not distinguished whether the screening at hospital was done prior to surgery or after. According to hospital guidelines, screening shall be completed before surgery, however, sometimes this is difficult to achieve.

Line 199-205 (results): At the hospital, healthcare professionals completely screened 123 (57.2 %) out of the 215 patients for being “at risk of malnutrition” or not, of which 96 patients (44.7 %) were screened preoperatively. Twenty-seven (12.6 %) of these patients did not complete the self-screening.

2. Thank you, we agree and has pointed this in our new conclusion:

Line 357-361 (conclusion): Completed by three out of four patients, preoperative self-screening in a home setting may be a feasible method to increase the number of elective surgical patients screened for being “at risk of malnutrition” before surgery. Of note, this may not be valid for all elective surgical patients and should therefore be viewed as a supplement and not a replacement for healthcare professionals’ screening.

*MAIN TEXT*

1. *Introduction (line 65) - font is different.*
2. *Statistics – state what is an adequate level of agreement for this study. See:* [*https://pubmed.ncbi.nlm.nih.gov/23092060/*](https://pubmed.ncbi.nlm.nih.gov/23092060/)
3. *Results - I think that there is maybe still a bit of confusion over what the study set out to achieve, more clarity is needed. If the main aim is to assess if self-screening increases number of patients screened for malnutrition risk prior to surgery, then this should be stated as a main outcome.*
4. *Results should open with and highlight the number (%) out of 215 that self-screened prior to surgery compared to the number (%) out of 215 that were screened in hospital prior to surgery. Currently this information reads more like descriptive stats rather than the main outcome and there is no direct comparison between numbers self-screened and numbers screened in hospital prior to surgery. This would allow you state that ‘self-screening improved malnutrition screening by X % compared to hospital screening prior to surgery.*
5. *Limitations – another limitation could be that there was a low number of patients who were identified as at risk of malnutrition.*
6. *Discussion – I felt that this started quite negatively by highlighting the main limitation of this study (missing screenings at hospital) – suggest moving this further down in the discussion. You could instead open more positively – this study highlights the importance of self-screening at home and demonstrates that self-screening is possible and increases numbers of patients screened for malnutrition risk prior to surgery. I think this should help to make your primary outcome come across better as the current level of agreement (secondary outcome) overshadows the main outcome.*

Authors response

1. Thank you. The font is now corrected.
2. Thank you for this point. We have carried out a new analysis, but the results did not change notably. We have not defined any threshold for an adequate level of agreement for this study, however based on the poor agreement regarding patients’ risk of malnutrition, we conclude that NRS 2002 is not our preferred tool for self-screening.

Line 222-224 (results): Comparing patients identified to be “at risk of malnutrition” by healthcare professionals with the self-reported screening tool and the patients’ belief regarding their own nutritional status, the degree of agreement was demonstrated to be *poor* for them both (κ = - 0.04 (95 % CI: -0.24, 0.16) and κ = - 0.03 (95 % CI: - 0.21, -0.15), respectively) (Table 3).

3. Aim is revised in the new manuscript:

Line 103-104 (introduction): The aim of the study was to investigate whether it is feasible for patients to answer questions related to nutritional risk screening in a home setting prior to hospital admission for surgery, and to investigate whether this could increase the number of patients being screened for risk of malnutrition. A secondary aim was to assess the agreement between patients’ perception of being at risk of malnutrition and healthcare professionals’ screening, using the NRS 2002 screening tool.

4. Thank you for this suggestion. We have now included the following sentence:

Line 204 (results): Self-screening improved malnutrition screening participation by 71.9 % compared to hospital screening prior to surgery, 165 (76.7 %) and 96 (44.7 %), respectively.

Line 252 (discussion): Three out of four patients completed the self-screening for nutritional risk and 70 % more patients were screened for risk of malnutrition prior to surgery by patients *and* health personnel compared to health personnel only.

5. Thank you for this comment. We have included this in our revised manuscript:

Line 340-341 (strengths and limitations): Moreover, there was a high number of patients identified to be at nutritional risk and non-responder patients.

6. Thanks a lot for this suggestion to a perfect start of our discussion:

Line 252: This study highlights the importance of self-screening at home and demonstrates that self-screening is possible and increases numbers of patients screened for malnutrition risk prior to surgery.

7. PLOS authors have the option to publish the peer review history of their article ([what does this mean?](https://journals.plos.org/plosone/s/editorial-and-peer-review-process#loc-peer-review-history)). If published, this will include your full peer review and any attached files.
If you choose “no”, your identity will remain anonymous but your review may still be made public.
**Do you want your identity to be public for this peer review?** For information about this choice, including consent withdrawal, please see our [Privacy Policy](https://www.plos.org/privacy-policy).

Reviewer #2: No

Reviewer #3: No

Reviewer #4: No

Reviewer #5: **Yes:** Debra Jones
