# Supplementary material for: Is self-screening for 'at risk of malnutrition' feasible in a home setting?
Source: PLoS One. 2024 Apr 16;19(4):e0299305. doi: 10.1371/journal.pone.0299305 (PMC11020964; doi:10.1371/journal.pone.0299305)
Supplement: Attachment — Submitted filename: second feedback for PLOS1 paper.docx [file pone.0299305.s007.docx]

**Title:**

title may flow better if ‘at’ removed. ‘Is self-screening for ‘risk of malnutrition’ feasible in a home setting?

**Abstract:**

line 24 -28: I felt that the intro for the abstract didn’t flow as well as it could – I have suggested some changes here: ‘Despite malnutrition **being** established as a well-known risk for post operative complications, the lack of **screening for nutritional risk** remains a challenge. The aim of this study was to investigate whether self-screening for nutritional risk prior to surgery **is feasible in a home setting** and **if it** will increase number of patients screened for nutritional risk, and secondly, to compare their screening results with **the ‘in-hospital assessments’ conducted by** healthcare professionals’’.

Line 30: Is it correct that ‘Feasibility’ has a capital letter? If its because this is the title of a separate study then maybe place quotation marks around this?

Line 31-32: Found this difficult to read. Suggest: As part of the checklist patients who were in a home setting and due to undergo surgery with the next 3 months or less, were asked questions……

Lines 39-43: The numbers still feel confusing to me – I had to go to the main text to understand these lines. 123 screened by HCPs seems like a redundant number with stating the breakdown between screening that occurred pre and post op. It is made more confusing by the fact that you are stating 96 were screened both at home and in hospital, which is the same number as the number screened in by HCPs pre surgery – this is the more important number to go in the abstract otherwise its unclear where the 69 comes from. Suggest removing line that states the overlap being 96 and replace with the information that out of 123 screened by HCPs, 96 were screened pre op so a difference of 69 and improvement by 71.9%. And as additional information it might be good to say that screening overall, when considering pre and post op screening, improved by 34.1% (n=42). And add this to main text results as well.

As a side note – To me it seems that the info about overlap between home screening and HCP screening (96) is only useful information for when you come to look at agreement, therefore, don’t entangle this information with the first and most important results. Only bring this information in when discussing agreement – I think this is why the numbers initially feel confusing.

**Introduction:**

Line 60: for use by healthcare professionals **for assessing** in-hospital…...

Line 72: remove first ‘at’ or change words in quotations to just ‘risk of malnutrition’ , Maybe consider keeping the first ‘at’ and removing the ‘at’ in quotation marks and then throughout, as perhaps not always necessary to say ‘at’ – as mentioned above for the title???

**Materials and methods**

Line 91: typo – should be ‘trial’. And consider changing next sentence to: ‘it was calculated that the present study would require a total of 300 elective surgical patients, across six clusters, over 14 months.

**Results – Screening performance:**

Consider comments mentioned above for the abstract. It might be clearer if the numbers are grouped together differently.

Discussion.

I think the first sentence is a different font?? And maybe tag onto the end of this that there is still a greater number of patient self-screened even when post-op screening by HCPs is taken into account.

Line 217: and the total **pre-operative** screening increased…...

Line228: change to: The problem of missing screening for “at risk of malnutrition” is well known both…

Line 255: add in – …and **are** responsible…...

Line 256: change to taking care of **patient risk** by….

Line 287: to overcome the barriers **of** low screening

Line 296: replace ‘Of note, if such,’ with ‘Therefore,’

The last few paragraphs of discussion repeat ‘of note’ quite a few times. Maybe consider changing some so its not so repetitive??

**Strengths and limitations**

Line 301: remove first mention of ‘being’

Line 306: typo – possible should be possibly.

Line 310-311: change to: **A** further **limitation** of the study **is** that

Line 312: prior to surgery, and **so** the….

**Clinical relevance**

Line 315: change to: This study demonstrates potential for elective patients to answer questions that can be used …...

Line 316: change to: Further development may allow for the identification of those patients ….

Line 321: for those who need **it.**

**Conclusion**

Line 323 - 324: consider changing to: in this study, the number of patients self-screening at home, preoperatively was higher than both in-hospital preoperative screening and total pre and postoperative in-hospital screening.

Line 329: remove ‘Even’
