# Supplementary material for: Is self-screening for 'at risk of malnutrition' feasible in a home setting?
Source: PLoS One. 2024 Apr 16;19(4):e0299305. doi: 10.1371/journal.pone.0299305 (PMC11020964; doi:10.1371/journal.pone.0299305)
Supplement: Attachment — Submitted filename: PLOS one second review.docx [file pone.0299305.s008.docx]

## Reviewer #3

*This manuscript aims to investigate the important question of how we can improve access to nutrition screening preoperatively to identify patients at risk of malnutrition and therefore at increased risk of postoperative complications. The manuscript contains some interesting data but needs major revisions to enable interpretation of the findings.*

Authors response

Thank you for your comment. The manuscript has been revised according to alle the comments from the reviewers and the interpretation of the findings is improved. This is described in detail in this response letter.

Thank you for taking the feedback on board. The changes made have increased the quality of the manuscript however there are still some issues with the methodology and interpretation of the findings. Revising these aspects would strengthen the paper and make it more relevant for the readers.

*Abstract*

1. *Line 28 - amount should be number of patients.*
2. *Line 45 - how many patients had both a healthcare professional and a self-screening completed? This is the most important number and should be reported.*
3. *What was the time difference between the screening tools being completed?*
4. *Line 47 - 48 - the conclusion does not reflect the results section. The agreement is stated as "poor".*

Authors response

1. “Amount of patients” is now changed to “*number* of patients” (line 48). “Amount of patients” is still used in the abstract introduction section.
2. The number of patients who had both a healthcare professional and a self-screening completed is 96 (67.9 %), this is pointed out in the results. This should also be included in the abstract. This is a key finding that relates to the second aim of “to compare their screening results with healthcare professionals’ in-hospital assessment”. The agreement statistics rely on this number. The abstract does not tell the reader what proportion of patients self-screened as at nutrition risk. Please add this result given the focus of the abstract is about self-screening. The abstract says that “at the hospital 123 (57.2%) were screened for being “at risk of malnutrition””. I find the phrase “at risk of malnutrition” very confusing. I first read this as 123 patients were at risk of malnutrition but on re-reading it I think you mean that 123 patients were screened for nutritional risk at the hospital b/c in the results section of the manuscript only 4% were screened as at risk of malnutrition. Suggest revising how this phrase is used.

Line 196-197: *Of these, 96 (67.9%) were screened by a healthcare professional at hospital.*

3. The self-screening was performed from 12 to two weeks before surgery. The health care workers screened the patients from two weeks and until the day before surgery. This variation depends on each surgical ward’s routine for the surgical admission and in some cases the screening was fulfilled after surgery. This is pointed out in the abstract and in the method section. Given the time difference between patient and healthcare professional screening the outcomes of screening cannot be compared. This patient group are likely to lose weight and have changes in food intake prior surgery which will impact upon the validity of the correlation statistics. This is a limitation of the study and should be included in the limitations section.

Line 55-60 (abstract): As part of the checklist, patients were up to 3 months preoperatively in a home-setting, asked questions to identify “at risk of malnutrition” in a moderated version of the Nutritional Risk Screening tool (NRS 2002).

Line 152-153 (method): Self-registration of the nutritional information as part of the PASC (14), “*at home”*, is hereby called the “self-reported screening tool” and was performed up to 3 months prior to surgery.

Line 155-158 (method): These results were compared with the healthcare professionals’ nutritional risk screening results which was performed at the hospital 2 weeks before and until the day before surgery and in some cases after surgery.

4. The abstract conclusion is now revised and in line with the results.

Line 72: Preoperatively self-screening in a home setting may be a feasible method to increase the number of elective surgical patients screened for risk of malnutrition. The patients and healthcare workers agreement regarding low body mass index, weight loss and reduced food intake was acceptable. The over-all agreement on the NRS 2002’s classification of “At risk of malnutrition” was *poor.* Is there sufficient power to run these statistics if only 1.9% of the hospital screened cohort were at risk of malnutrition? NRS 2002 may not be an appropriate tool for nutritional risk self-screening for surgical patients at home. Also, the self-screening aspect did not use the validated for of the NRS 2002 because one of the questions was not included.

*Methods*

1. *Line 87 - how was this sample size calculated? Please include further details.*
2. *Line 96 - 98 - were patients recruited while an inpatient or at an outpatient clinic appointment? It is not clear. Why did health professionals not complete the nutrition screening at this point in time rather than when patients were admitted for surgery?*
3. *Please add further details about the NRS 2002 questions and the timing of screening.*
4. *Lines 105 to 107 - how did calculate BMI? Was this based on self-reported weight and height? Did they have to use tables on paper to work this out? Please include the exact screening tool questions that were used in this study in a table or figure or supplementary information.*
5. *Lines 113- 117. It is unclear if the scoring system for "at risk of malnutrition" was changed given patients did not complete all questions. Please include a comment about whether this is an acceptable use of the screening tool.*
6. *Section starting Line 101. When did patients complete the tool? If a patient completes the tool 12 weeks prior to the health professional completing the tool the inter-rater agreement cannot be compared b/c they are not assessing the same state. It is likely that changes in weight and food intake will occur during this period.*
7. *Line 119 - 120 - has this question (... the patients were asked on the same paper form if they thought they were “at risk of malnutrition” (“yes”/”no”)) been used in previous studies? Please provide evidence to support its inclusion or at least justify why it has been included.*

Authors response

1. In our revised manuscript it is clearer that our study was part of a larger project, and the power calculating was performed for this larger study. We have changed the paper in ref 14 to clarify this.

Line 115-116 (methods): The power calculation for this study was based on the PASC trail protocol. It was calculated a need of a total of 300, elective surgical patients, across six clusters over 14 months (14).

2. Participants were recruited by study personnel or health care workers after receiving the date for surgery, either at the outpatient clinic appointment or by mail. The health care personnel performed the screening according to the hospital’s routines which is now explained in the manuscript:

Line 122-125 (methods): The study participants were recruited by study personnel in cooperation with the nurses and surgeons at each ward. This was done either at the outpatient clinic appointment or by letter in the mail, after the patient had received a date for surgery.

Line 155-157 (methods): ... the healthcare professionals’ nutritional risk screening results which was performed at the hospital 2 weeks before and until the day before surgery and in some cases after surgery.

3. See authors response in the abstract section, number 3 (over).
4. Thank you for the opportunity to clarify this. We have re-vised Figure 1, showing both the screening procedure and the exact self-screening tool, which is the nutrition questions from NRS2002 included in the patient surgery checklist. Based on patients answering the questions in Figure 1, study personnel calculated BMI and the NRS2002-scores. This is explained in line 142-149. The revised figure 1 was not available to review.

Line 144-151 (methods): NRS 2002 is designed for use by healthcare personnel. Some adjustments were made for patients’ self-screening with NRS2002; First, the initial question regarding critical illness and the final screening questions that quantify the severity of their disease to be moderate or severe were left out, since patients in such cases would not be at home but hospitalized. Secondly, the screening tool was re-design to be more patient user-friendly, see Figure 1. Finally, based on the patients’ answers to the questions and their age, study personnel calculated the BMI and, NRS 2002 scores, and categorized the patients to be “at risk of malnutrition” (≥ 3 scores) or not (< 3 scores).

5. The modification of NRS 2002 is discussed as a weakness of the study:

Line 352-358 (strengths and limitations): In addition, the modifications to NRS 2002 to customize the screening tool for patients’ self-screening may have affected its reliability. Of note, similar modifications have also been suggested to self-reporting screening by using MUST (10).

6. See authors response to the abstract section number 3 (over). We cannot exclude the possibility of bias due to this timeline, as discussed as a weakness of the study in line 345-347. I cannot see this included in the revised version I received to review.

Line 355-358 (strengths and limitations): Further limitations of the study include that healthcare professionals most often screened the patients after surgery, whereas the patients screened themselves prior to surgery, and that the response rate may induce a selection bias.

7. The question whether the patient thought they were “at risk of malnutrition” is a question in the surgical patient’s checklist (PASC) and not a part of the screening tool NRS 2002. We are not aware of others using this question. We think the patient’s perception regarding their nutritional status is of interest, even this single question is of minor value. We have some qualitative data analyses in progress to explore this. It would be helpful to include that data in this manuscript to strength the rationale for using this more explorative question in the manuscript.

*Statistical methods
This section does not detail which kappa measure of agreement was used. Please review accordingly. Assumption 5 of Cohen's kappa was not met - "Assumption #5: The same two raters are used to judge all observations (e.g., patients). This has been referred to as having fixed or unique raters. If different raters were used for each observation (e.g., patient), Cohen's kappa is not the appropriate test to use. However, in this latter case, you could use Fleiss' kappa instead, which allows randomly chosen raters for each observation (e.g., patient)."*

Authors response

Thank you for this comment. We agree with the reviewer and have performed a new analysis by Fleiss multi rater Kappa with overall agreement. This method assess the [reliability of agreement](https://en.wikipedia.org/wiki/Inter-rater_reliability) between a fixed number of [raters](https://en.wikipedia.org/wiki/Rater) when assigning [categorical ratings](https://en.wikipedia.org/wiki/Categorical_rating) to a number of items or classifying items (Wiki). This is changed in the result section, in Table 2, 3 and 4, and outlined in the statistics section. Further, we had removed the analyses of positive degree of agreement, negative degree of agreement, the prevalence index, and the bias index.

*Results*

1. *Line 169 - were the patients who believed they were at risk of malnutrition the same patients who were identified as at risk of malnutrition on the NRS2002? This is important to know.*
2. *Line 171-172 "All these patients were screened after surgery". The use of the screening tool after surgery is not detailed in the methods section. Please make it clear in the. methods section when HPs and patients completed the screening tool.*
3. *Line 192 - be aware of over interpreting the statistics. The number of patients identified at risk of malnutrition was only 5% of the population and the agreement statistic is based on a small sample of 9 versus 11 screening tool results.*

Authors response

1. This important result is presented in detail in the following section in the manuscripts: Agreement regarding being “at risk of malnutrition” or not (Line 225-234). The agreement between being and believe being “at risk of malnutrition” or not is outlined in the results line 230-231 and visualized in detail in Table 4.

Line 233-234: The agreement between the self-reported screening tool, and the patients’ belief regarding being “at risk of malnutrition” was fair (κ = 0.27 (95 % CI: -0.12, 0.30)) (Table 4).

2. Thank you for this comment. In cases where the routine screening failed (n=27), the patients were screened after surgery. This is now clarified in the manuscript.

Line 155-157 (methods): … the healthcare professionals’ nutritional risk screening results which was performed at the hospital 2 weeks before and until the day before surgery and in some cases after surgery.

3. Thank you for this important remark. This study was performed to get experience with the PASC checklist and nutrition risk self-screening. Based on our experiences, we cannot recommend the NRS2002 have chosen another screening tool for the main project, and we will recommend other to do the same, even the sample size is small.

*Discussion*

1. *Line 230 - this needs a reference please.*
2. *Line 255 - it is not clear what the "high prevalence indices" is referring to.*
3. *Line 260 - 262 - these results are not clearly presented in the results section. Please revise accordingly.*
4. *Line 264 - please add further details about the time difference to the results section. This is very important as timing of risk of malnutrition prior to surgery dictates the type of intervention that could be provided to reduce risk preoperatively.*
5. *Line 278 to 279 - yes very important point!*
6. *Line 284 - the time delay here is also important. For example, screening by health professionals at the time of booking surgery date would be a more accurate method to compare with self-screening at time of booking.*

Authors response

1. We do not have a reference to this sentence and has therefor revised it:

Line 287 (discussion): Several factors may affect the patient’s appetite before surgery, such as the underlying cause of surgery, as well as fear of surgery and/or pain or illness.

2. We agree about the prevalence index and have deleted this from the text and tables.
3. We think this is clearly presented in Table 2, however vi understand that “… the criteria in the self-reporting screening tool, “might be unclear and have changed this.

Line 324 (discussion): “… the questions in the self-reporting screening tool, …

4. See authors response to **Reviewer #3 the abstract section number 3 (over)**.
5. Thank you.
6. Yes, indeed we agree. Hopefully, patients will routinely be screened for malnutrition at the time of booking surgery date in the future. And hopefully might this paper contribute to increased attention to this possibility.
