# Supplementary material for: Is self-screening for 'at risk of malnutrition' feasible in a home setting?
Source: PLoS One. 2024 Apr 16;19(4):e0299305. doi: 10.1371/journal.pone.0299305 (PMC11020964; doi:10.1371/journal.pone.0299305)
Supplement: Attachment — Submitted filename: Response to reviewers.docx [file pone.0299305.s009.docx]

## Response letter

## Editor

*The reviewers have raised a number of concerns that need attention, including reference to comments provided at the last round of revision to which no response was provided. They request additional information on methodological aspects of the study and revisions to the wording used to increase clarity.*

Authors response

We apologize that some corrections were not visible and will ensure that they all will be so this time. The methodology is revised and thoroughly explained in the revised Figure 1, 2 and 3, in the Material and Method section and the Result section. Also, the Discussion is revised, and the Conclusion is rewritten. We hope you will find this satisfactory.

## Reviewer #3

*Thank you for taking the feedback on board. The changes made have increased the quality of the manuscript, however there are still some issues with the methodology and interpretation of the findings. Revising these aspects would strengthen the paper and make it more relevant for the readers.*

1. *Line 28 - amount should be number of patients.*
2. *Line 45 - how many patients had both a healthcare professional and a self-screening completed? This is the most important number and should be reported.*
3. *What was the time difference between the screening tools being completed?*
4. *Line 47 - 48 - the conclusion does not reflect the results section. The agreement is stated as "poor".*
5. *The revised figure 1 was not available to review.*
6. *Section starting Line 101. When did patients complete the tool? If a patient completes the tool 12 weeks prior to the health professional completing the tool the inter-rater agreement cannot be compared b/c they are not assessing the same state. It is likely that changes in weight and food intake will occur during this period.*
7. *Line 119 - 120 - has this question (... the patients were asked on the same paper form if they thought they were “at risk of malnutrition” (“yes”/”no”)) been used in previous studies? Please provide evidence to support its inclusion or at least justify why it has been included.*

Authors response

Thank you for your valuable comments.

1. The “amount” is changed to “number”

Line 25-27: The aim of this study was to investigate whether self-screening for nutritional risk prior to surgery is feasible in a home setting and if it will increase number of patients screened for nutritional risk …

Line 54-56: Preoperatively self-screening in a home setting may be a feasible method to increase the number of elective surgical patients screened for risk of malnutrition.

2. We have revised the abstract, results, and Figure 3, to show clearly that 96 patients were both self-screened at home and by health care personnel at hospital.

Line 41-43: A total of 123 (57.2 %) patients were screened in-hospital, of whom 96 (44.7%) prior to surgery and 96 (44.7 %) were screened both at hospital (pre- and post-surgery) and at home.

Line 195-196: Out of 164 (76.7 %) patients completing the self-reported screening tool at home, 96 (67.9 %) were screened pre- or postoperatively by a healthcare professional at hospital.

Figure 3. Flowchart of the study population.

3. The timeline is better explained in the abstract, method section, and figure 2, and discussed in the strengths and limitations.

Line 32-35: This checklist included a self-reported screening tool based on the Nutritional Risk Screening tool (NRS 2002) to identify “at risk of malnutrition” in patients that will undergo surgery the next 3 months or less.

Line 106-108: Participants were prospectively recruited when scheduled to surgery by the hospital within a period of 2 to 12 weeks before surgery, depending on the patients’ severity of disease and need for surgery. Patients returned their completed PASC checklists, including nutritional risk screening questions, before discharge or by mail.

Line 316-318: Also, due to the hospital’s routines, in some cases it could have been up to three months between the self-screening performed at home and the routine screening performed at hospital.

Line 318-319: During the weeks prior to surgery, patients may experience weight loss and changes in their food intake that will impact upon the validity of the correlation statistics.

Figure 2. The screening procedure.

4. Thank you for your comment, we see that we have not be clear enough in our conclusion and have done following amended to the text:

Line 338-346: Conclusion. In this study, three out of four surgical patients performed self-screening at home, thus the preoperative screening rate improved with 70%, which shows that patients are able to and willing to perform self-screening at home. This is an important step towards involving patients in their own surgical safety as patients self-screening is a feasible and important method to increase the number of elective surgical patients screened for risk of malnutrition before surgery. The next step is to investigate other self-screening tools and to give nutritional support to the patients that identify themselves with nutritional risk to improve nutritional status and thus patient safety prior to surgery.

5. We apologize and will ensure that all the figures and corrections will be better visible this time.
6. Regarding the timeline, see Authors response to pt. 3, above.
7. The question whether the patients thought they were “at risk of malnutrition” was added to raise their awareness of the nutritional status prior to surgery. We wanted to investigate if patients have knowledge regarding whether low BMI, low food intake and weight loss may be risk factors prior to surgery. This single question is not a part of NRS2002 and is thus of minor value in this paper, and, as far as we know, this is not investigated earlier. However, we think the patient’s perception regarding their nutritional status is of interest and have some qualitative data analyses in progress to explore this.

Line 135-137: To raise awareness of nutritional status prior to surgery, the patients were asked on the same paper form if they thought they were “at risk of malnutrition” (“yes”/”no”).

## Reviewer #4

1. *I reviewed the article "Practicability of self-screening for "at risk of malnutrition" among surgical patients in a home setting.” The study's primary purpose is to investigate and compare the agreement rates between preoperatively measured self-reported malnutrition screening at home by patients and at the hospital by nurses. Although the study has a good design and presentation, some major methodological points make the study lower quality.*

*Missing data when choosing the patients in the study is much more than expected, which may cause a bias in the analyses.*

Authors response

The manuscript has been through a major revision to improve the quality. The methodology and discussion are revised, as also figure 1, 2 and 3.

It is a fear number of patients who performed self-screening at home and were screened in-hospital. Our findings have important clinical relevance, as expressed in the new conclusion (see above). However, the missing data regarding the prevalence of patients “at risk of malnutrition” is a weakness of our study. The degree of agreement between the patient and health care worker is weak, which may be due to the missing values, the timeline, or that NRS2002 is not developed for self-screening, as discussed in the strengths and limitations.

Line 313-320. The main limitation is the low number of patients identified to be “at risk of malnutrition” at home, and at hospital, since this decreases the possibility of a reliable comparison of the screening results between patients and healthcare professionals. Also, due to the hospital’s routines, in some cases it could have been up to three months between the self-screening performed at home and the routinely screening performed at hospital. During the weeks prior to surgery, patients may experience weight loss and changes in their food intake that will impact upon the validity of the correlation statistics.

2. *The hypothesis and purpose of the study needed to be well documented in the paper. I could not understand what this work would add to this area. Preoperatively self-measured malnutrition rate should be like measurements in the hospital. However, kappa analysis results show shallow coefficient scores.*

Authors response

Malnutrition is present in 30% of the patients in Norwegian hospitals, leading to increased risk for adverse clinical outcomes and even mortality. During the last decades our hospital has introduced nutritional risk screening. Early identification of patients at risk of malnutrition makes it possible to prevent severe malnutrition by proper assessment and tailored nutritional care. Unfortunately, nearly half of the surgical patients are still not screened for malnutrition risk, and the screening rate is difficult to increase. Therefore, pre-operative self-screening at home is an opportunity to increase the screening rate and thus to improve nutritional status prior to surgery in cases where this is worthy for patients’ safety.

We wanted to investigate to which degree the patients could perform self-screening at home. This was done 2-12 weeks before hospitalization for surgery. During the weeks before surgery, patients might experience reduced food intake and weight loss that may explain why the preoperatively self-measured malnutrition rate may differ from the measurements in the hospital.

The high number of patients performing self-screening at home is an important opportunity to improve the patient’s safety. This is even more high-lighted in the results and conclusion and brings clarity to the hypothesis and purpose.

Line 184-187: Self-screening improved malnutrition screening participation by 71.9 % compared to hospital screening prior to surgery (165 (76.7 %) and 96 (44.7 %), respectively) and by 34.1 % compared to pre- and postoperative screening at the hospital 165 (76.7%) and 123 (57.2 %), respectively).

Line 341-346: This is an important step towards involving patients in their own surgical safety as patients self-screening is a feasible and important method to increase the number of elective surgical patients screened for risk of malnutrition before surgery. The next step is to investigate other self-screening tools and to give nutritional support to the patients that identify themselves with nutritional risk to improve nutritional status and thus patient safety prior to surgery.

## Reviewer #5

***Title:***

*title may flow better if ‘at’ removed. ‘Is self-screening for ‘risk of malnutrition’ feasible in a home setting?*

Authors response

We agree that the title flows better without “at”, however, according to the European consensus on nutritional terminology (<https://pubmed.ncbi.nlm.nih.gov/30181091/>) it should not be removed.

***Abstract:***

*line 24 -28: I felt that the intro for the abstract didn’t flow as well as it could – I have suggested some changes here: ‘Despite malnutrition* ***being*** *established as a well-known risk for post operative* *complications, the lack of* ***screening for nutritional risk*** *remains a challenge. The aim of this study was to investigate whether self-screening for nutritional risk prior to surgery* ***is feasible in a home setting*** *and* ***if it*** *will increase number of patients screened for nutritional risk, and secondly, to compare their screening results with* ***the ‘in-hospital assessments’ conducted by*** *healthcare professionals’’.*

*Line 30: Is it correct that ‘Feasibility’ has a capital letter? If its because this is the title of a separate study then maybe place quotation marks around this?*

*Line 31-32: Found this difficult to read. Suggest: As part of the checklist patients who were in a home setting and due to undergo surgery with the next 3 months or less, were asked questions……*

*Lines 39-43: The numbers still feel confusing to me – I had to go to the main text to understand these lines. 123 screened by HCPs seems like a redundant number with stating the breakdown between screening that occurred pre and post op. It is made more confusing by the fact that you are stating 96 were screened both at home and in hospital, which is the same number as the number screened in by HCPs pre surgery – this is the more important number to go in the abstract otherwise its unclear where the 69 comes from. Suggest removing line that states the overlap being 96 and replace with the information that out of 123 screened by HCPs, 96 were screened pre op so a difference of 69 and improvement by 71.9%. And as additional information it might be good to say that screening overall, when considering pre and post op screening, improved by 34.1% (n=42). And add this to main text results as well.*

*As a side note – To me it seems that the info about overlap between home screening and HCP screening (96) is only useful information for when you come to look at agreement, therefore, don’t entangle this information with the first and most important results. Only bring this information in when discussing agreement – I think this is why the numbers initially feel confusing.*

Authors response

Thank you very much, we appreciate your valuable feedback on our article. All of them have been a beneficial help to improve the manuscript. We agree in all these constructive comments and have revised the manuscript according to your points. The “new” abstract is as following:

Line 24-56: Abstract

Introduction: Despite malnutrition **being** established as a well-known risk for postoperative complications, the lack of **screening for nutritional risk** remains a challenge. The aim of this study was to investigate whether self-screening for nutritional risk prior to surgery **is feasible in a home setting** and if it will increase number of patients screened for nutritional risk, and secondly, to compare their screening results with **the “in-hospital assessments” conducted by** healthcare professionals.

Materials and methods: This was a prospective study involving patients from six randomly selected surgical wards at two Norwegian hospitals as a part of the **“Feasibility study of implementing the surgical Patient Safety Checklist the (PASC)”. This checklist included a self-reported screening tool based on the Nutritional Risk Screening tool (NRS 2002) to identify “risk of malnutrition” in patients that will undergo surgery the next 3 months or less**. The original screening tool (NRS 2002) was used as a standard routine to identify “at risk of malnutrition” by healthcare professionals at hospital. The interrater reliability between these results was investigated using Fleiss multi rater Kappa with overall agreement and reported with Landis and Koch’s grading system (*poor*, *slight*, *fair*, *moderate*, *substantial*, and *almost perfect*).

**Results: Out of 215 surgical patients in the home setting, 164 (76.7 %) patients completed the self-reported screening tool. A total of 123 (57.2 %) patients were screened in-hospital, of whom 96 (44.7%) prior to surgery and 96 (44.7 %) were screened both at hospital (pre and post-surgery) and at home.** **Self-screening at home improved malnutrition screening participation by 71.9 % compared to hospital screening prior to surgery (165 (76.7 %) and 96 (44.7 %), respectively) and by 34.1 % compared to pre- and postoperative screening at the hospital 165 (76.7%) and 123 (57.2 %), respectively). The degree of agreement between patients identified to be “at risk of malnutrition” by the self-reported screening tool and healthcare professionals was *poor* (κ = - 0.04 (95 % CI: -0.24, 0.16), however, the degrees of agreement between the patients and healthcare professionals answers to the initial NRS 2002 questions “low BMI”, “weight loss”, and “reduced food intake” were *almost perfect* (κ = 1.00 (95% CI: 0,82, 1.18)), *moderate* (κ = 0.55 (95% CI: 0.34, 0.75)), and *slight* (κ = 0.08 (95% CI: - 0.10, 0.25) respectively.**

Conclusions: Three out of four patients completed the self-screening form and the preoperative screening rate improved with 70%. Preoperatively self-screening in a home setting may be a feasible method to increase the number of elective surgical patients screened for risk of malnutrition.

***Introduction:***

1. *Line 60: for use by healthcare professionals* ***for assessing*** *in-hospital…...*
2. *Line 72: remove first ‘at’ or change words in quotations to just ‘risk of malnutrition’ , Maybe consider keeping the first ‘at’ and removing the ‘at’ in quotation marks and then throughout, as perhaps not always necessary to say ‘at’ – as mentioned above for the title???*

Authors response

1. We agree. The following change is done:

Line 63: … for use by healthcare professionals **for assessing** in-hospital…...

2. We agree that the little word «at» is a bit confusing. According to the European consensus, we keep “at” and have removed **being** several places to achieve a better flow, as her:

Line 69-70: Society for Clinical Nutrition and Metabolism expert group recommend that patients be screened for “at risk of malnutrition” at least 10 days before surgery.

***Materials and methods***

1. *Line 91: typo – should be ‘trial’. And consider changing the next sentence to: ‘it was calculated that the present study would require a total of 300 elective surgical patients, across six clusters, over 14 months.*

Authors response

1. This is now re-written.

Line 93-95: “The power calculation was based on the PASC trail protocol. It was calculated that the present study would require a total of 300 elective surgical patients, across six clusters, over 14 months (14).”

***Results – Screening performance:***

*Consider the comments mentioned above for the abstract. It might be clearer if the numbers are grouped together differently.*

Authors response

We agree and have revised according to this comment.

Line 184-187: Self-screening improved malnutrition screening participation by 71.9 % compared to in-hospital screening prior to surgery (165 (76.7 %) and 96 (44.7 %), respectively) and by 34.1 % compared to pre- and postoperative screening at the hospital 165 (76.7%) and 123 (57.2 %), respectively).

Line 195-196: Out of 164 (76.7 %) patients completing the self-reported screening tool at home, 96 (67.9 %) were screened pre- or postoperatively by a healthcare professional at hospital.

**Discussion.**

*I think the first sentence is a different font??*

*And maybe tag onto the end of this that there is still a greater number of patients self-screened even when post-op screening by HCPs is taken into account.*

*Line 217: and the total* ***pre-operative*** *screening increased…...*

*Line228: change to: The problem of missing screening for “at risk of malnutrition” is well known both…*

*Line 255: add in – …and* ***are*** *responsible…...*

*Line 256: change to taking care of* ***patient risk*** *by….*

*Line 287: to overcome the barriers* ***of*** *low screening.*

*Line 296: replace ‘Of note, if such,’ with ‘Therefore,’*

Authors response

Thank you very much, we agree. The font is corrected, the introduction to the discussion is improved according to your suggestion, and all your comments are taken into account.

Discussion, line 226-231: This study highlights the importance of self-screening at home and demonstrates that self-screening is possible and increases numbers of patients screened for malnutrition risk prior to surgery. Three out of four patients completed the self-screening for nutritional risk. The number of patients self-screening at home, preoperatively was more than 70% higher than in-hospital preoperative screening, and 34% higher than total pre- and postoperative in-hospital screening.

Line 243: The problem of missing screening for “at risk of malnutrition” is well-known both nationally and internationally.

Line 270-272: The healthcare personnel **are** trained for screening and are responsible for taking care of the **patients’ risk** by assessment, nutritional treatment and finally to communicate information regarding nutritional therapy to next level of care.

Line 300: In an attempt to overcome the barriers **of** low screening performance for “at risk of malnutrition”, …

Line 309-310: **Therefore,** it is important to facilitate a treatment offer for those who identify themselves as being “at risk of malnutrition”.

*The last few paragraphs of discussion repeat ‘of note’ quite a few times. Maybe consider changing some so its not so repetitive??*

Authors comments

We agree and has revised:

Line 294: These patients were screened by the healthcare professionals after the surgery.

Line 309-310: Therefore, it is important to facilitate a treatment offer for those who identify themselves as being “at risk of malnutrition”.

***Strengths and limitations***

*Line 301: remove first mention of ‘being’*

Author response

We agree and has revised:

Line 313-316: The main limitation is the low number of patients identified to be “at risk of malnutrition” at home, and at hospital, since this decreases the possibility of a reliable comparison of the screening results between patients and healthcare professionals.

*Line 306: typo – possible should be possibly.*

Author response

Line 320-323: Previously published analysis showed no difference between genders on responding but demonstrated that the central community hospital had a higher number of non-responders, **possibly** indicating that patients with more complex surgery and medical conditions were the ones who participated in the study.

*Line 310-311: change to:* ***A*** *further* ***limitation*** *of the study* ***is*** *that*

*Line 312: prior to surgery, and* ***so*** *the….*

Author response

Line 326-328: **A** further **limitation** of the study **is** that healthcare professionals may screen the patients after surgery, whereas the patients screened themselves prior to surgery, and **so** we cannot exclude the possibility of a screening bias.

***Clinical relevance***

*Line 315: change to: This study demonstrates potential for elective patients to answer questions that can be used …...*

*Line 316: change to: Further development may allow for the identification of those patients*

*Line 321: for those who need* ***it.***

Author response

We agree and have made the following changes:

Line 331-337: **This study demonstrated potential for elective patients to answer questions that can be used** to assess their nutritional status prior to surgery in a home setting. **Further development may allow for the identification of those patients** who are “at risk of malnutrition” prior to hospitalization and utilize the waiting time prior to surgery to safeguard and improve their nutritional status. Further investigation should include the electronic formats that may improve the accuracy and ease of use of self-screening, and include an associated treatment offer for those who need **it.**

**Conclusion**

*Line 323 - 324: consider changing to: in this study, the number of patients self-screening at home, preoperatively was higher than both in-hospital preoperative screening and total pre and postoperative in-hospital screening.*

*Line 329: remove ‘Even’*

Authors response

We agree and have re-written the conclusion where “even” is removed, se above.

Line 339-346: In this study, three out of four surgical patients performed self-screening at home, thus the preoperative screening rate improved with 70%, which shows that patients are able to and willing to perform self-screening at home. This is an important step towards involving patients in their own surgical safety as patients self-screening is a feasible and important method to increase the number of elective surgical patients screened for risk of malnutrition before surgery. The next step is to investigate other self-screening tools and to give nutritional support to the patients that identify themselves with nutritional risk to improve nutritional status and thus patient safety prior to surgery.
